# The Lysine Demethylase KDM4C Is an Oncogenic Driver and Regulates ERK Activity in KRAS-Mutant Pancreatic Ductal Adenocarcinoma

**DOI:** 10.1158/2767-9764.CRC-25-0278

**Published:** 2026-01-30

**Authors:** Menna-t-Allah Shaheen, Sarah Dhebat, Kimal I. Rajapakshe, Bidyut Ghosh, Benson Chellakkan Selvanesan, Shariq S. Ansari, Cara L. Haymaker, Dorsay Sadeghian, Huamin Wang, Ching-Fei Li, Haoqiang Ying, Anirban Maitra

**Affiliations:** 1 Department of Translational Molecular Pathology, The University of Texas MD Anderson Cancer Center, Houston, Texas.; 2 Sheikh Ahmed Center for Pancreatic Cancer Research, The University of Texas MD Anderson Cancer Center, Houston, Texas.; 3 Department of Pathology and Immunology, Baylor College of Medicine, Houston, Texas.; 4 Department of Anatomical Pathology, The University of Texas MD Anderson Cancer Center, Houston, Texas.; 5 Department of Molecular and Cellular Oncology, The University of Texas MD Anderson Cancer Center, Houston, Texas.

## Abstract

Deregulation of proteins involved in chromatin regulation is common in pancreatic ductal adenocarcinoma (PDAC). Lysine demethylase 4C (KDM4C) is one of the chromatin-modifying proteins frequently overexpressed across multiple solid cancers and is linked to chromatin instability, increased cell proliferation, and enhanced stem cell–like behavior. We observed upregulation of KDM4C protein in a panel of human PDAC cell lines and patient samples compared with nonneoplastic controls. CRISPR/Cas9-mediated deletion of *KDM4C* in human and murine PDAC cells reduced proliferation, clonogenicity, and increased survival of orthotopically implanted murine PDAC allografts. Transcriptomic and proteomic analyses revealed that loss of KDM4C in both human and murine PDAC cell lines was associated with the reduction of activated phospho-ERK, a pivotal effector downstream of mutant RAS. Using proximity labeling, we identified the histone deacetylase SIRT1 as a novel interacting protein with KDM4C via the latter’s Tudor reader domain. SIRT1-mediated deacetylation leads to repression of downstream targets, including the dual specificity phosphatase DUSP2, which is known to inactivate ERK via dephosphorylation. *In vitro* propagation of *KDM4C*-null PDAC lines eventually led to adaptation and restitution of ERK signaling, with rescue of the KDM4C loss induced growth suppression. To bypass this adaptive phenomenon, we tested a preclinical pan-KDM4 inhibitor TACH107 and confirmed its efficacy in *in vitro* and *in vivo* PDAC models. Our studies identify KDM4C as an oncogenic molecule that sustains ERK signaling in KRAS-mutant PDAC and can be broadly targeted via small-molecule inhibitors.

**Significance:**

Our data suggests that KDM4C is a novel regulator of ERK signaling, the main effector pathway downstream of mutant RAS. This is the first demonstration linking the requirement of sustained KDM4 activity to ERK signaling in cancer and presents an opportunity to leverage this oncogenic pathway for therapeutic intervention.

## Introduction

Pancreatic ductal adenocarcinoma (PDAC) is a highly recalcitrant neoplasm and is anticipated to become the second leading cause of cancer-related deaths in the United States by 2040 (1). PDAC exhibits significant resistance to standard-of-care chemotherapeutic regimens, and despite extensive research efforts, the 5-year survival rate remains at a mere 12% (2). Greater than 90% of PDAC harbor mutant KRAS, and although agents targeting KRAS have been developed recently, both inherent and acquired resistance to these inhibitors limit their efficacy (3). Besides genetic mutations, it is estimated that approximately a quarter of PDAC cases have disrupted histone modification enzymes, and 40% demonstrate dysregulation of epigenetic regulators (4). Numerous small-molecule inhibitors targeting epigenetic enzymes are available, presenting a significant yet largely unexplored therapeutic opportunity in PDAC.

The histone lysine-specific demethylase 4C (KDM4C), initially identified as “gene amplified in squamous cell carcinoma 1” (GASC1), is part of a group of five histone demethylases (KDM4A-E) that target the histone marks H3K9me3/me2 and H3K36me3/me2 and are conserved across different species (5, 6). KDM4A-C share structural similarities and exhibit both overlapping and unique functions. They are composed of three protein domains: the Jumonji (JMJ) domain, which is responsible for catalytic activity, followed by two reader domains: the plant homeodomain (PHD) and Tudor domains (5). Physiologically KDM4C is expressed early in embryonic development and in embryonic stem cells to maintain low H3K9me3 levels and open chromatin (7). KDM4C has been frequently found to be overexpressed in a variety of solid and liquid tumors, including breast, lung, colon, prostate, and esophageal cancers and lymphomas (6, 8–14). However, until now, KDM4C has not been studied in the context of pancreatic cancer, and the molecular mechanisms underlying KDM4C oncogenic properties are not well understood.

We hypothesized that KDM4C overexpression in PDAC is essential for cell proliferation by epigenetically regulating essential cell signaling pathways. In this study, using a variety of cross-species and orthogonal approaches (namely, human and murine cell lines, archival samples, and allograft models), we demonstrate that KDM4C is overexpressed in PDAC and is associated with more aggressive disease biology. Interestingly, we demonstrate that KDM4C activity is required for sustained activation of ERK, a pivotal effector downstream of mutant KRAS, and restitution of ERK activity in *KDM4C*-null cells is associated with rescue of growth suppression. Our data also identify a novel KDM4C-interacting partner, the histone deacetylase (HDAC) SIRT1, and demonstrates that SIRT1-mediated deacetylation and repression of the ERK phosphatase DUSP2 could underline the previously unreported link between KDM4C and the RAS pathway. Finally, we highlight the potential of KDM4C as a candidate therapeutic target that could increase the efficacy of chemotherapy in patients with PDAC.

## Materials and Methods

### Cell lines and culture

Human PDAC cell lines AsPC1 (RRID: CVCL_0152) and Pa03C (RRID: CVCL_E301) and murine PDAC cell line derived from *KRAS*^*LSL-G12D/+*^, *Trp53*^*LSL-R172H/+*^, *Pdx1-Cre* (KPC) mice KPC mT4 (Dr. David Tuveson, Cold Spring Harbor Laboratories; ref. 15) as well as HPNE (RRID: CVCL_C466) and HEK293 cell lines (RRID: CVCL_0045) were used. HPNE cells were maintained in 75% DMEM without glucose (Sigma-Aldrich, cat. #D5030) with 2 mmol/L L-glutamine and 1.5 g/L sodium bicarbonate, 25% Medium M3 Base (Incell Corp., cat. #M300F-500), 5% fetal bovine serum (FBS; Thermo Fisher Scientific, cat. #10082147), 10 ng/mL human recombinant EGF, 5.5 mmol/L D-glucose (1 g/L), and 750 ng/mL puromycin. AsPC1 was maintained in RPMI 1640 (Thermo Fisher Scientific, cat. #11875093), supplemented with 10% FBS, penicillin (100 U/mL), and streptomycin (100 μg/mL). Pa03C, KPC-mT4, and HEK293 cell lines were maintained in DMEM (Thermo Fisher Scientific, cat. #11965092). The cell lines were routinely tested and confirmed to be *Mycoplasma*-free using MycoAlert Mycoplasma Detection Kit (Lonza, cat. #LT07-318). AsPC1 (cat. #CRL-1682), HPNE (cat. #CRL-4023), and HEK293 (cat. #CRL-1573) cells were purchased from the American Tissue Culture Collection (ATCC). Pa03C is obtained from the previous Maitra Lab at Johns Hopkins University, and KPC-mT4 is from Dr. David Tuveson, Cold Spring Harbor Laboratories. AsPC1, Pa03C, and HPNE cell lines were authenticated by short tandem repeat (STR) analysis DNA fingerprinting using PowerPlex 16 HS Kit (Promega, cat. #DC2100) at the MD Anderson Cytogenetics and Cell Authentication Core (CCAC). The STR profiles were compared with online search databases (DSMZ/ATCC/JCRB/RIKEN) along with the CCAC database. The STR profiles matched known DNA fingerprints. KPC-mT4 and HEK293 cell lines were not authenticated. KPC-mT4 genotype was confirmed by MiniMUGA genotyping array v0009 (Neogen, cat. #553). Protein lysates for Pa02C, Pa09C, and Pa16C were obtained from the previous Maitra Lab at Johns Hopkins University, and MDA28 protein lysate was obtained from H. Ying (MDACC).

### Pancreatic tissue microarrays

We used previously described tissue microarrays (TMA), comprised of 187 PDAC cases (16). This study was approved by the Institutional Review Board of MD Anderson Cancer Center (MDACC). The study approval number is Protocol: Lab05-0854. This protocol has a waiver for informed consent. From each case, two punches (1.5 mm) from separate tumor areas (T1 and T2) and one punch (1.5 mm) from the normal pancreas (N) were included. After immunohistochemistry (IHC) staining with anti-KDM4C antibody, the samples were evaluated by a pathologist (D.S.) for both the intensity and percentage of stained cells in a semi-quantitative way. Intensity: 0: no staining/1: weak/2: moderate/3: strong. Proportion: 1: less than 10%/2: 10% to 50%/3: 50% to 75%/4: more than 75%. The final immunoscore was calculated by adding the intensity score to the proportion score according to the Allred method (17). The average immunoscore score from the tumor area was divided by the immunoscore from the matched normal area for each case. The log_2_ ratio was then used for survival analysis, with a threshold log_2_ ratio of 0.32.

### CRISPR-mediated deletion of *KDM4C*

The genomic sequence for the *KDM4C* gene was downloaded from the UCSC genome browser (RRID: SCR_005780). Two single-guide RNA (sgRNA) cassettes targeting exon2 of human *KDM4C* and two sgRNA cassettes targeting murine *KDM4C* were designed using the CRISPR design tool (http://crispr.mit.edu; ref. 18) and cloned into pspCas9 plasmid vector (Addgene, cat. #62988, RRID: Addgene_62988; Supplementary Table S1). Human PDAC cell line AsPC1 and murine PDAC cell line KPC-mT4 were transfected with respective sgRNA Cas9 plasmids and selected under 2 μg/mL puromycin for 5 days before the cells were serially diluted into 96-well plates to form single colony clones. Knockout (KO) clones were then expanded, and successful gene editing was confirmed using T7-endonuclease assay. Biallelic deletion of *KDM4C* was validated using *in vitro* transcription (Guide-it Genotype Confirmation Kit, Takara, cat. #632635), and frameshift mutation was confirmed by sequencing.

### Western blot analysis

Cell pellets were lysed in Mammalian protein extraction reagent (M-PER, Thermo Fisher Scientific, cat. #78501) with the addition of protease/phosphatase inhibitor cocktail. The extracted protein was quantified using Bradford assay. Electrophoresis was performed to separate proteins in 4% to 12% of Bis–Tris gels (Thermo Fisher Scientific). The samples were subsequently transferred onto a nitrocellulose membrane. After 1 hour of blocking in 5% milk (RRID: ABT), membranes were incubated with either anti-KDM4C (1:500 dilution; Santa Cruz Biotechnology, cat. #sc-515767, RRID: AB_3068588), anti–pERK1/2 (1:1,000; Cell Signaling Technology, cat. #9101, RRID: AB_331646), anti–ERK1/2 (1:1,000; Cell Signaling Technology, cat. #9102, RRID: AB_330744), anti-KDM4A (1:1,000; Cell Signaling Technology, cat. #5328, RRID: AB_10828595), anti-KDM4B (1:1,000; Cell Signaling Technology, cat. #8639, RRID: AB_11140642), anti-KDM4D (1:1,000; Santa Cruz Biotechnology, cat. #sc-393750, RRID: AB_3674229), anti-KDM4E (1:1,000, Thermo Fisher Scientific, cat. #PA5-71128, RRID: AB_2691349), anti-DUSP2 (1:300, Santa Cruz Biotechnology, cat. #sc-32776, RRID: AB_2094883), or horseradish peroxidase (HRP)-conjugated anti–β-actin (1:10,000; Santa Cruz Biotechnology, cat. #sc-47778, RRID: AB_626632) primary antibodies at 4°C overnight. Depending on the host for the primary antibody, the blots would then be incubated with an HRP-conjugated anti-rabbit IgG (Cell Signaling Technology, cat. #7074, RRID: AB_2099233) or anti-mouse IgG secondary antibody (Cell Signaling Technology, cat. #7076, RRID: AB_330924) for at least 1 hour at room temperature. Clarity ECL Western blotting substrates (Bio-Rad, cat. #1705061) were used to visualize the blots. Full images for all Western blot panels are included in Supplementary Fig. S7.

### Colony formation assay

To assess colony formation ability, 1,000 cells were seeded in triplicates in six-well cell culture–treated plates and cultured for 6 to 7 days or until optimal confluency was reached. Media were aspirated, and the colonies were washed with PBS, and fixed using 4% formalin for 30 minutes. To visualize colonies, the plates were stained with 0.05% crystal violet for 60 minutes. After washing and drying, the plates were imaged, and the wells analyzed for colony count using ImageJ software (RRID: SCR_003070).

### Cell proliferation assay

Cell proliferation was monitored using the IncuCyte Live Cell Imaging System. Briefly, 1,000 cells in 100 mL of media were seeded per well in a 96-well culture plate in triplicates per condition. Phase-contrast images were captured every 6 hours and were transformed into percentage confluency. Six images were collected per well per time point and are used for confluence analysis.

### Orthotopic injection

All procedures were reviewed and approved by the Institutional Animal Care and Use Committee (IACUC) at MDACC under protocol number 00001222-RN03. Immunodeficient nude mice (Nu/J, cat. #002019, RRID: IMSR_JAX:002019) or C57BL/6 (B6/J mice, cat. #000664, RRID: IMSR_JAX:000664) were purchased from The Jackson Laboratory and used for orthotopic injections. The detailed procedure was described previously (19). Briefly, 1 × 10^5^ of *KDM4C* KO KPC-mT4 or wild-type (WT) parental control cells were suspended in 50 mL of 1:1 media and Matrigel mixture. Male and female mice at approximately 8 weeks of age were anesthetized with isoflurane, and the cells were injected orthotopically into the tail of the pancreas following approved IACUC protocol. Buprenorphine ER was used as an analgesic before the procedure and 48 hours after surgery. The mice were sacrificed after the specified number of days, and the pancreas tissues were collected.

### Quantitative real-time PCR and RNA sequencing

Total RNA was extracted from cell pellets using the RNeasy Mini Kit (Qiagen, cat. #74104), following the manufacturer’s protocol. A total of 200 ng RNA was used to produce cDNA by reverse transcription. Gene expression levels were normalized to GAPDH and quantified by real-time RT-PCR using the δ-δ Ct method. The primers were purchased from Integrated DNA Technologies and the sequences included in Supplementary Table S2.

### Bulk RNA sequencing data analysis

Bulk RNA sequencing (RNA-seq) was performed in triplicate on a NextSeq500 (Illumina). Quality of the paired-end reads were assessed with FastQC (v0.11.8). Poor quality reads were filtered with seqtk (v1.3-r106) before aligning the high-quality reads to primary assembly GRCh38 of the human genome sequence using the STAR aligner (v2.6.1d). Sequencing reads were mapped using STAR against the human genome (UCSC hg38), gene-level quantification was performed using featureCounts (v v2.0.1), and raw counts were imported into DESeq2 for statistical analysis to compare gene expressions between groups. Genes with false discovery rate < 0.05 and fold change (FC) ≥1 (or ≤0.5) were considered as highly differentiated genes. Ingenuity pathway analysis (IPA) was performed by importing the highly differentiated genes gene into QIAGEN IPA (QIAGEN Inc.). Gene Set Enrichment Analysis (GSEA) was carried out using the HALLMARK collection from MSigDB to identify the gene sets enriched in each experimental group.

### Reverse phase protein array

Briefly, 1 × 10^6^ cells per sample were collected and washed twice with PBS, then lysed and quantified. Samples were prepared in triplicates and submitted to the Functional Proteomics core at MDACC for detection and analysis, as described (20). Differentially expressed proteins between groups were identified using a parametric *t* test on normalized reverse phase protein array (RPPA) data. Significance was achieved at *P* < 0.05 and FC >1.25 (or <1/1.25).

### RNAi and short hairpin RNA knockdown

Two independent short hairpin RNAs (shRNA) targeting human *KDM4C* were obtained from Horizon Discovery and cloned into pLKO.1 vector (Addgene, cat. #10878, RRID: Addgene_10878; Supplementary Table S3). Lentivirus was produced in HEK293 cells by transfecting the vectors with psPAX2 (Addgene, cat. #12260, RRID: Addgene_12260) and pMD2.G (Addgene, cat. #12259, RRID: Addgene_12259) packaging system. Virus particles were collected after 48 and 72 hours and concentrated using a Lenti-X concentrator (Takara, cat. #631232). Cells were transduced with the lentivirus carrying shRNA or empty vector as a control using polybrene and then selected under 2 μg/mL puromycin for 5 days. DUSP2 siRNA knockdown was performed using the DUSP2 Human siRNA Oligo Duplex (Locus ID 1844; Origene, cat. #SR301296) following the manufacturer’s instructions.

### Cytometry by time of flight

*Sample preparation*: The PDAC tumor tissues were homogenized to produce single-cell suspension. 3 × 10^6^ cells were subsequently blocked with anti-mouse Fc block and stained with cell ID cisplatin for live–dead marker. Furthermore, the samples were barcoded with CD45 antibody and combined. The pooled samples were stained with the antibody cocktails obtained from the MD Anderson Flow cytometry and Imaging Core facility and titrated before use using standard protocol from Standard BioTools. After staining, the cells were fixed in 1.6% paraformaldehyde and stained with Cell ID Intercalator-Ir and resuspended in Maxpar water, and the samples were run on cytometry by time of flight (CyTOF) Helios instrument. For data analysis, the CD45^+^ samples were de-barcoded using CyTOF software (Fluidigm Corporation) and converted into individual fcs files and further analysis was done in FlowJo (version 10.10.0, RRID: SCR_008520). The samples were cleaned up and down from dead cells, debris, and doublets, downsampled to 1 × 10^4^ cells per replicate (*n* = 3 per sample), and concatenated, and clustering algorithms Phenograph and FlowSOM were used to identify the cell types. The CyTOF antibody panel used for this study encompassed markers for the detection of T cells and myeloid immune cell populations (Supplementary Table S4).

### TACH107 studies

The pan-KDM4 inhibitor TACH107 [tool compound for TACH101 (21), Tachyon Therapeutics] was dissolved in DMSO and stored as 10 mmol/L aliquots in light-protective vials at −80°C for *in vitro* studies. For *in vivo* experiments, TACH107 powder was dissolved in PBS and polyethylene glycol 400 (PEG 400) in 1:1 ration, and the pH was adjusted to 9 using 10N NaOH and litmus paper strips. 1 × 10^5^ cells from the mouse PDAC cell line KPC-mT4 was suspended in 1:1 media: Matrigel solution and then inoculated into the pancreas of 8-week-old female B6 mice as previously described. The mice were randomized into two groups: a TACH107-treated group and a vehicle control group. The treatment group received 80 mg/kg TACH107 in 200 μL volume by oral gavage once daily for 28 days. The survival of mice was recorded, and the tumors of moribund or euthanized mice were harvested.

### Viability assay

MTT Cell Proliferation Assay Kit (Abcam, cat. #ab211091) was used according to the manufacturer’s protocol. Briefly, 5,000 cells per well were seeded in a 96-well plate in a volume of 100 μL. The following day, cells were treated with specified doses of the panKDM4 inhibitor TACH107 (Tachyon) or DMSO for 48 hours. MTT assay reagent was then added and incubated for 3 hours at 37°C before adding the MTT solvent. The absorbance was read at 590 nm.

### Cellular thermal shift assay

To verify pan-KDM4 inhibitor TACH107 binding to target proteins, we performed cellular thermal shift assay (CETSA; 22). In this method, target binding is validated by observing the increase in thermal stabilization of ligand-bound target proteins. Briefly, 1 × 10^6^ AsPC1 cells were plated in a 10-cm culture dish and treated with pan-KDM4 inhibitor TACH107 or DMSO control for 60 minutes. Cells were then harvested and heated in a Bio-Rad gradient thermocycler for 3 minutes at temperatures between 42°C to 47°C to generate a melting curve. The samples were then lysed and loaded for Western blotting.

### IHC and immunofluorescence

Pancreatic tissues were collected at the specified time points and fixed in 4% paraformaldehyde. Paraffin-embedded sections were stained as previously described (23). Primary antibody incubations were carried out overnight at 4°C using the following antibodies: rat anti–E-cadherin (1:100, Thermo Fisher Scientific, cat. #14-3249-82, RRID: AB_1210458), rabbit anti-pERK (1:100, Cell Signaling Technology, cat. #9101, RRID: AB_331646), rabbit anti-KDM4C (1:100, Novus, cat. #NBP1-49600, RRID: AB_10011699), mouse anti-H3K9Me3 (1:100, Active Motif, cat. #61013, RRID: AB_2687870), and mouse anti-HA tag (1:100, Thermo Fisher Scientific, cat. #26183-A488, RRID: AB_2610624). For IHC, HRP-conjugated secondary antibodies were sourced from Vector Laboratories: anti-Rabbit IgG (cat. #MP-7401, RRID: AB_2336529) and anti-Mouse IgG (cat. #MP-7452, RRID: AB_2744550). 3-3′ Diaminobenzidine tetrahydrochloride (Vector Laboratories, cat. #SK-4100, RRID: AB_2336382) was used as a chromogen. Brightfield images were acquired using an Olympus light microscope. For immunofluorescence (IF), 4 × 10^4^ cells were seeded into 8-well chamber slides, incubated overnight, and then treated with DMSO or 1 μmol/L TACH107 for 24 hours. Secondary antibodies were obtained from Abcam and used at 1:300 dilution; anti-rat IgG (Cy3, cat. #ab98416, RRID: AB_10673341), anti-mouse IgG (FITC, cat. #ab6785, RRID: AB_955241), anti-mouse IgG (Cy3, cat. #ab97035, RRID: AB_10680176), and anti-rabbit IgG (FITC, cat. #ab6717, RRID: AB_955238). Samples were mounted with fluorescence mounting media with DAPI (Vector Laboratories, cat. #H-1200-10) and left to dry overnight. Slides were imaged on the Olympus confocal microscope and quantified with ImageJ.

### Proximity labeling using miniTurboID

We followed the protocol described by Cho and colleagues (24). Briefly, the coding region for *KDM4C* was cloned into 3xHA-miniTurbo-NLS_pCDNA3 plasmid (RRID: Addgene_107172), which contains nuclear localization signal and HA-tag to produce “KDM4C-miniTurboID” fusion construct. The fusion protein expression, localization, and biotinylating activity was verified after it was transfected into AsPC1 cells by IF and Western blotting (Supplementary Fig. S6A and S6B). Biotinylated proteins were enriched using Streptavidin Magnetic Beads (Thermo Fisher Scientific, cat. #88817), and the washed and dried beads were submitted to the Proteomics Core at UT Southwestern Medical Center, Dallas, for mass spectrometry analysis. The overall abundance of all proteins with high confidence, supported by two or more unique detected peptides, was imported into the R package preprocessCore (v1.66.0) for normalization. The normalized data were then transformed to log_2_ and used for a ratio-metric approach as described in the original protocol. Three different controls were used along with the experimental “KDM4C-miniTurboID plus biotin” sample: an Untransfected control, a miniTurboID control, and a KDM4C-miniTurboID without biotin control. A list of high-confidence hits was generated based on the following criteria: KDM4C-miniTurboID + Biotin/Untransfected control >0, KDM4C-miniTurboID + Biotin/KDM4C-miniTurboID without biotin >0, and KDM4C-miniTurboID + Biotin/TurboID >0. The identified proteome was subsequently analyzed using Cytoscape (RRID: SCR_003032). A stricter criterion using KDM4C-miniTurboID + Biotin/TurboID >1 was used to identify the top list of interactors.

### Co-immunoprecipitation

To validate protein binding with endogenous KDM4C, PDAC cells were lysed on ice in nondenaturing lysis buffer (50 mmol/L Tris–HCl pH 7.5, 150 mmol/L NaCl, 1% NP-40, 1 mmol/L EDTA) with protease and phosphatase inhibitors. Lysates were precleared by incubating with Protein A/G agarose beads (Thermo Fisher Scientific, cat. #20421) for 45 minutes. Then 1 mg of total protein in 500 μL volume was incubated with 1 μg respective antibodies: anti-SIRT1 (1:100, Abcam, cat. #ab110304, RRID: AB_10864359), anti-PARP1 (1:100, Cell Signaling Technology, cat. #9542, RRID: AB_2160739), anti-ZNF148 (1:100, Sigma-Aldrich, cat. #HPA001656, RRID: AB_1079995), anti-KDM4C (1:100, Novus, cat. #NBP1-49600, RRID: AB_10011699), or anti-IgG control (1:100, Thermo Fisher Scientific, cat. #14-4714-82, RRID: AB_470111), overnight at 4°C, with rotation, whereas 25 μg of lysate was reserved as input control. Next day, the lysates were incubated with 40 μL Protein A/G agarose slurry for 2 hours at room temperature. The beads were then collected and washed three times with lysis buffer, and proteins were eluted for detection by immunoblotting.

### KDM4C domain deletion constructs

We generated domain-specific deletion mutants of KDM4C by inverse PCR. Primers were designed to delete specific domain by using an online primer design tool from Takara. All PCR reactions were carried out using high-fidelity PCR enzyme Pfu DNA polymerase under the following conditions: initial denaturation at 95°C/3′, followed by 18 cycles at 95°C/45′ (denaturation), 62°C/1′ (annealing), and 68°C/2′ per Kb (Extension) with a final extension at 68°C for 10′. Following the PCR, the reaction mix was treated with Dpn1 for 2 hours at 37°C to destroy the methylated parental template. The PCR reaction was directly transformed, DNA was prepared, and sequence verified to confirm the deletion.

### Histone chromatin immunoprecipitation sequencing

Chromatin immunoprecipitation (ChIP) assays were performed at the MDACC Epigenomics Profiling Core as described previously with some modifications (25, 26). Briefly, AsPC1 parental and *KDM4C* KO cells were cross-linked with 1% formaldehyde for 10 minutes at room temperature, followed by quenching with 125 mmol/L glycine for 5 minutes. Chromatin was prepared by sonication to generate DNA fragments ranging from 200 to 600 bp. ChIP was performed overnight using DiaMag Protein A–conjugated antibodies against H3K27ac (Abcam, cat. #ab4729, RRID: AB_2118291) and H3K36me3 (Abcam, cat. #ab9050, RRID: AB_306966). Immunocomplexes were collected the following day using DynaMag and then washed and reverse cross-linked overnight, followed by DNA extraction. Libraries were prepared using NEB Ultra II DNA Library Prep Kit (New England Biolabs, cat. #E7645), according to the manufacturer’s protocol, and subjected to next-generation sequencing to obtain ∼30 million 75 nt single reads per sample. The sequencing was performed on NextSeq500 High Output 75ntSR flow cell.

### ChIP-seq data analysis

Raw FASTQ files were processed using the ENCODE ChIP-seq pipeline2 to assess the data quality and perform initial processing. First, the raw reads were trimmed with Trimmomatic (v0.39) to remove the adapter sequences. The trimmed reads were then mapped to the GRCh38 genome using Bowtie2 (v2.2.6). Mapped reads were further processed with samtools (v1.7) to remove unmapped reads, retain properly paired reads, remove duplicates, and remove multi-mapped reads (MAPQ <30) reads. The peaks for the aligned data were performed with MACS2 (v2.2.7.1), and differential peak calling was analyzed with R package DiffBind (v3.4.11) for differential binding affinity analysis.

### Statistical analysis

GraphPad Prism software (version 10.0, GraphPad Software, RRID: SCR_002798) was used to generate plots and analyses. Statistical significance is based on *P* < 0.05 and was calculated by Student *t* test, one-way analysis of variance (ANOVA), and Pearson correlation coefficient tests, where appropriate.

## Results

### KDM4C is differentially upregulated in pancreatic cancer and correlates with poor prognosis

**Figure 1.**
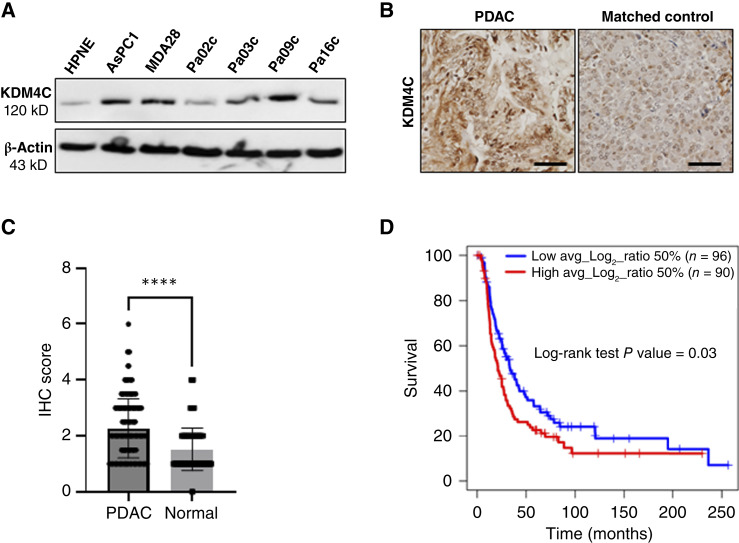
KDM4C is differentially upregulated in pancreatic cancer and correlates with poor prognosis. **A,** Western blots show human PDAC cell lines overexpress KDM4C compared with the noncancerous pancreatic epithelial cell line HPNE. **B,** Representative photomicrographs of KDM4C expression by IHC on TMAs of archival PDAC samples demonstrates upregulation of KDM4C in neoplastic cells compared with matched nonneoplastic pancreatic tissues. **C,** Bar graph showing the mean and distribution of the immunoscores of KDM4C expression in 187 cases of PDAC and matched normal pancreas. The score difference between the groups was assessed for statistical significance via paired *t* test; ****, *P* ≤ 0.0001. **D,** Kaplan–Meier survival plot showing increased survival of patients with low-KDM4C expressing PDAC (*n* = 96) compared with high-expressing tumors (*n* = 90) from the TMA dataset (*P* = 0.03). To determine KDM4C expression, we used PDAC TMAs, comprised of 187 PDAC cases. From each case, two punches (1.5 mm) from separate tumor areas (T1 and T2) and one punch (1.5 mm) from the normal pancreas (N) were included. After IHC staining with anti-KDM4C antibody, the samples were evaluated by a pathologist for both the intensity and percentage of stained cells in a semi-quantitative way. Intensity: 0: no staining/1: weak/2: moderate/3: strong. Proportion: 1: less than 10%/2: 10% to 50%/3: 50% to 75%/4: more than 75%. The final immunoscore was calculated by adding the intensity score to the proportion score according to the Allred method. The average immunoscore score from the tumor area was divided by the immunoscore from the matched normal area for each case. The log_2_ ratio was then used for survival analysis, with a threshold log_2_ ratio of 0.32.

To determine the protein expression levels of KDM4C in PDAC, we screened a panel of six human PDAC cell lines, AsPC1, MDA28, Pa02C, Pa03C, Pa09C, and Pa16C, against the nonneoplastic pancreatic epithelial cell line HPNE. Five of six cell lines (∼83%) showed increased KDM4C protein expression compared with the control (Fig. 1A). Next, we assessed KDM4C protein levels by IHC in archival human PDAC TMAs comprised of tissue cores from 187 resected PDAC cases with matched normal pancreas. We found that KDM4C expression levels were significantly higher in PDAC compared with normal (Fig. 1B and C). To determine the clinical relevance of KDM4C in patients with PDAC, we compared the survival of patients with high versus low KDM4C-expressing tumors and found that higher KDM4C expression significantly correlates with shorter overall survival, *P* = 0.03 (Fig. 1D). Our results demonstrate that KDM4C is overexpressed in PDAC cell lines and in patient tumor tissues, and higher expression correlates with poor prognosis in patients.

### CRISPR-mediated *KDM4C* deletion attenuates PDAC growth *in vitro* and *in vivo*

**Figure 2.**
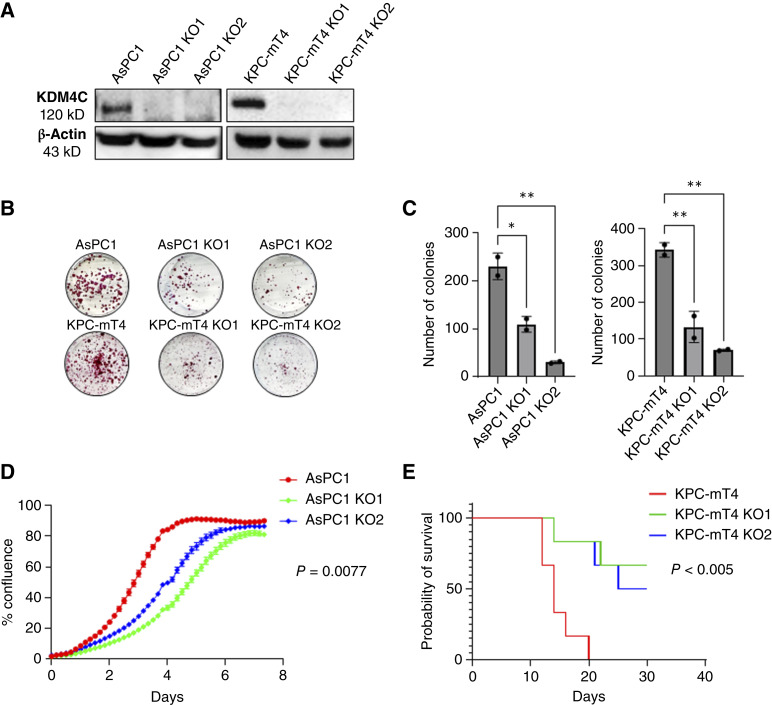
CRISPR-mediated *KDM4C* deletion attenuates PDAC growth *in vitro* and *in vivo*. **A,** Western blot showing *KDM4C* depletion in selected AsPC1 and KPC-mT4 *KDM4C*-null clones. **B,** Colony formation assay (CFA): representative wells comparing AsPC1 cells to *KDM4C* KO isogenic cells show reduced colony formation ability in the absence of KDM4C. **C,** Quantification of CFA by ImageJ shows a significant reduction in the number of colonies in *KDM4C* KO cells compared with *KDM4C* WT cells; *P* value was determined by one-way ANOVA test; *, *P* ≤ 0.05; **, *P* ≤ 0.01. **D,** Proliferation assay measured through % confluence over time by the Incucyte Live Imaging system shows decreased proliferation in the absence of KDM4C, *P* value determined by one-way ANOVA test. **E,** Kaplan–Meier survival plot showing increased survival of B6 mice orthotopically transplanted with *KDM4C* KO KPC-mT4 cells compared with those transplanted with *KDM4C* WT KPC-mT4 (*n* = 6 per arm).

As KDM4C is elevated in pancreatic cancer, we postulated that KDM4C might play a role in sustaining the growth of PDAC cells. To explore the requirement for this protein in PDAC, we used CRISPR/Cas9 to delete endogenous *KDM4C* and isolated *KDM4C*-null clones from human and mouse PDAC cell lines (AsPC1 and KPC-mT4, respectively; Fig. 2A). Our findings revealed that the absence of KDM4C diminishes the colony-forming capacity and proliferation of PDAC cells *in vitro* (Fig. 2B–D). To assess whether this effect persists *in vivo*, we orthotopically injected *KDM4C* WT parental or two independent *KDM4C* KO clones from KPC-mT4 cells into the pancreas of syngeneic immunocompetent B6 mice. The results showed that mice receiving *KDM4C*-deleted clones had extended survival (Fig. 2E). To address the specificity of KDM4C function within the KRAS-driven PDAC context, we used CRISPR/Cas9 to knock out *KDM4C* in HPNE cells and observed reduced proliferation in *KDM4C* KO HPNE cells compared with parental controls; however, the effect was notably less pronounced than in PDAC cells (Supplementary Fig. S1A and S1B). These findings imply that elevated KDM4C expression contributes to PDAC cell proliferation and growth both *in vitro* and *in vivo*.

### KDM4C expression correlates with ERK activity

**Figure 3.**
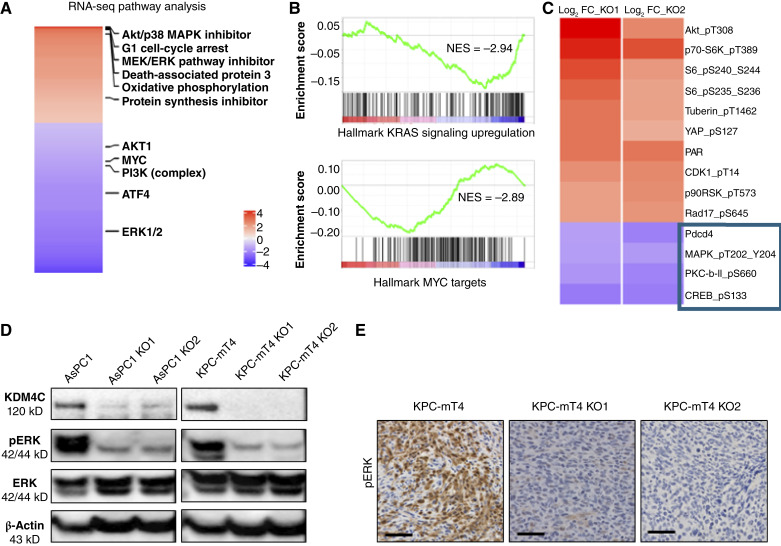
KDM4C expression correlates with ERK activity. **A,** Top enriched IPA pathways from RNA-seq data from *KDM4C* KO vs. WT AsPC1 are cell-cycle arrest, inhibition of MEK/ERK, protein synthesis inhibition, and increased oxidative phosphorylation and apoptosis, whereas ATF4, MYC, and ERK1/2 are among the top downregulated networks. **B,** GSEA plot from RNA-seq data showing downregulated KRAS and MYC signatures in *KDM4C*-depleted cells. **C,** RPPA data demonstrate downregulation of the ERK pathway in two different *KDM4C*-null clones. **D,** Western blot validating downregulation of ERK phosphorylation in human PDAC cell line AsPC1 and in mouse PDAC cell line (KPC-mT4) *KDM4C* KO clones. **E,** Representative micrographs of pERK expression by IHC in PDAC tumors harvested at day 30 from mice injected with PDAC cell line KPC-mT4 demonstrates downregulation of pERK in the *KDM4C* KO clones compared with untreated KPC-mT4 (Scale bar, 100 μm).

To identify KDM4C effector pathways that contribute to the observed effects on PDAC growth, we performed RNA-seq studies on *KDM4C* KO clone versus *KDM4C* WT AsPC1 cells (PCA analysis and heatmap of differentially expressed genes are in Supplementary Fig. S2A and S2B). Notably, pathway analysis of the differentially expressed gene set in the absence of KDM4C revealed a mitogen-activated protein kinase inhibitor–like signature as one of the top enriched pathways, in addition to G1 cell-cycle arrest, protein synthesis inhibition, and increased oxidative phosphorylation and apoptosis (Fig. 3A). On the other hand, top downregulated pathways in the absence of KDM4C included ERK1/2, ATF4, and MYC signaling. Furthermore, GSEA showed downregulated signatures for KRAS signaling and MYC, which cooperates with RAS in orchestrating PDAC progression (27, 28) reiterating the role of KDM4C in regulating pathways pivotal to PDAC tumorigenesis (Fig. 3B).

To explore the signaling alterations in *KDM4C*-deficient cells, we conducted a RPPA analysis on two *KDM4C* KO clones and compared them with *KDM4C* WT control AsPC1 cells. Notably, RPPA revealed 32 proteins with differential expression in the KO clones relative to the WT control. Among these, the most downregulated phospho-proteins were pERK, pCREB, and pPKC, indicating that KDM4C is involved in activating the ERK signaling pathway (Fig. 3C). To confirm these results, we utilized human PDAC cell line AsPC1 and mouse PDAC cell line KPC-mT4 and two distinct *KDM4C* KO clones from each. We consistently observed that active pERK protein levels were downregulated in the absence of KDM4C, whereas the total ERK levels remained unchanged. This suggests that KDM4C does not directly regulate the expression of these proteins (or transcripts) but rather influences their activation (Fig. 3D). Additionally, tumors harvested from *KDM4C* KO KPC-mT4 inoculated mice showed lower pERK expression compared with those from *KDM4C* WT KPC-mT4 tumors (Fig. 3E). Furthermore, *KDM4C*-null KPC-mT4 cells exhibited reduced sensitivity to ERK inhibition compared with WT cells, suggesting that the protumorigenic function of KDM4C in PDAC is ERK-dependent (Supplementary Fig. S2C). We conclude that KDM4C is an essential regulator of ERK activity in PDAC cells.

### Adapted late-passage *KDM4C*-null clones restore ERK signaling and rescue *in vitro* phenotype

**Figure 4.**
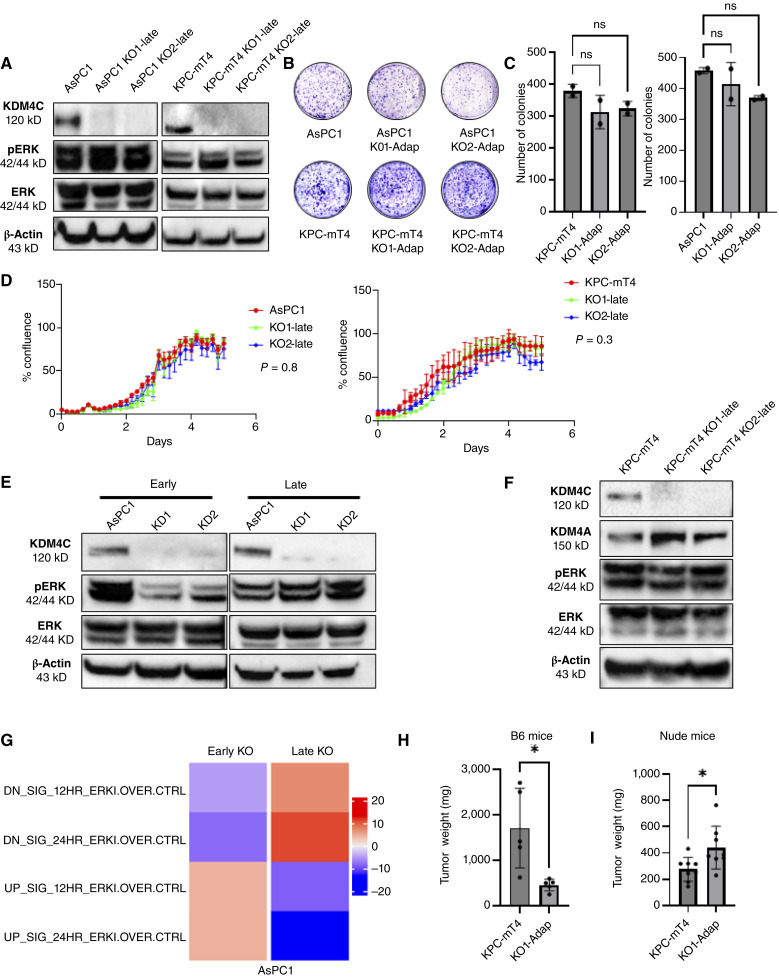
Compensatory upregulation of KDM4A restores cell-intrinsic ERK signaling in *KDM4C*-null cells but not immune surveillance. **A,** Western blot panel shows that late-passage *KDM4C* KO clones of both AsPC1 and KPC-mT4 (KO-late) restored pERK levels. **B–D,** ERK reactivation rescues the reduction in colony formation and proliferation in both AsPC1 and KPC-mT4 KO cells observed in earlier passages. Bar plots show number of colonies calculated by ImageJ. For each sample, two wells have been analyzed, and the statistical significance is calculated using one-way ANOVA. For both AsPC1 and KPC-mT4 cells, the difference between parental and adapted *KDM4C* KO clones was not significant. **E,** Lentiviral shRNA knockdown of *KDM4C* recapitulates the adaptation to KDM4C depletion in adapted cells. Western blot panel compares early-passage (day 7 after transduction) and late-passage (day 40 after transduction) in AsPC1 cells. Late-passage *KDM4C *knockdown cells have adapted to the loss of KDM4C and restored ERK activation. **F,** Western blot validating KDM4A upregulation in adapted KPC-mT4 *KDM4C* KO clones. **G,** RNA-seq results from AsPC1 early-passage *KDM4C* KO cells correlate with the ERK inhibitor (ERKi) transcriptome signature, whereas late-passage adapted cells are inversely correlated. **H** and **I,** Bar graphs showing significant reduction in the tumor growth of adapted KPC-mT4 *KDM4C* KO cells when transplanted into the immunocompetent B6 mice compared with *KDM4C* WT control (*n* = 5), whereas the reverse is observed when the same cells are transplanted into immune-compromised athymic mice (*n* = 8), significance determined by unpaired *t* test; *, *P* ≤ 0.05.

Upon passaging *KDM4C*-null cells, we observed that the late-passage cells (KO-late) of both human and murine origin restored ERK signaling as indicated by the increase in phosphorylated ERK levels (Fig. 4A). Restored ERK activation was accompanied with rescue of the colony formation and proliferation phenotypes that were initially observed with KDM4C loss (Fig. 4B–D). As this finding was retrospectively investigated, we sought to prospectively validate and time the adaptation of PDAC cells to the loss of function of KDM4C using shRNA knockdown. Passaging and collecting the cells at 7-day intervals, we found that PDAC cells begin to restitute ERK signaling as early as 40 days after *KDM4C* knockdown (Fig. 4E). To investigate the mechanisms underlining this adaptation, we performed RPPA study on adapted *KDM4C* KO KPC-mT4 cells (KO1-late) and their parental KPC-mT4 WT controls and found that several candidate proteins, including the KDM family member, KDM4A, were overexpressed in the adapted cells (Supplementary Fig. S3). The KDM4A upregulation was confirmed by Western blot (Fig. 4F), and this KDM family member emerged as a potential candidate in the absence of KDM4C to restore ERK signaling, but alternative adaptive mechanisms such as RTK activation may also be involved as suggested by the RPPA data. We also interrogated the transcriptome-based ERK inhibition signature as described by Klomp and colleagues (29) in RNA-seq data from the early-passage nonadapted KDM4C-null cells versus the later-passage adapted lines. Interestingly, we found that the early- passage nonadapted *KDM4C*-null cells exhibit an ERK inhibition signature that is reversed in the later-passage adapted state (Fig. 4G).

As shown previously (Fig. 2E), loss of KDM4C in early passage cells is associated with reduced tumor growth *in vivo*. Therefore, we investigated whether the later-passage *KDM4C* KO cells would exhibit rescue of their tumor growth rates *in vivo* due to adaptation. We orthotopically transplanted KPC-mT4 *KDM4C* WT or adapted KO1 cells into the pancreas of B6 mice, and the resulting tumors were collected after 30 days. Surprisingly, the adapted *KDM4C* KO cells continued to generate significantly smaller tumors than their WT counterparts (Fig. 4H). We then repeated the experiment in immune-deficient athymic mice, in which tumor growth was not only rescued in the *KDM4C* KO adapted cohort but the resulting allografts were significantly larger than those in the WT control (Fig. 4I), likely reflecting the compensatory effects of ERK hyperactivation. These observations led us to hypothesize that endogenous KDM4C also inhibits immune surveillance in PDAC and that the cell-intrinsic restitution of ERK activity in the setting of KDM4C loss is not able to completely revert these non–cell-autonomous effects. To test this hypothesis, we performed CyTOF assay from freshly harvested pancreatic tumors of immunocompetent B6 mice orthotopically injected with murine parental KPC-mT4 and adapted *KDM4C* KO cells. The immunophenotypic analysis revealed several antitumorigenic immune cell alterations that persist in the adapted *KDM4C*-null lines, including increased populations of B cells, CD8 T cells, and mast cells, along with decreased neutrophils, tumor-associated macrophages, and monocytic myeloid-derived suppressor cells (Supplementary Fig. S4A and S4B). Collectively, these data illustrate the importance of KDM4C in sustaining ERK activation in pancreatic cancer and suggest that PDAC cells eventually adapt to the loss of KDM4C by cell-intrinsic restitution of ERK signaling through alternative mechanisms. Nonetheless, the favorable impact of KDM4C loss on the pancreatic cancer immune microenvironment persists, even in the face of cell-intrinsic rescue of ERK signaling.

### Pan-KDM4 inhibitor TACH107 reduces proliferation and colony formation in PDAC cell lines *in vitro* and increases survival *in vivo*

**Figure 5.**
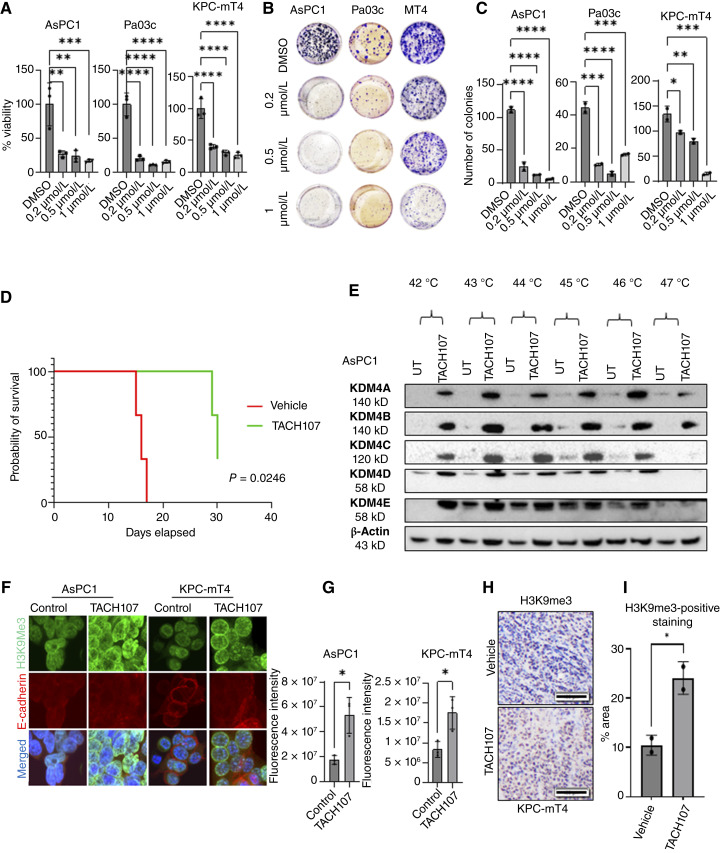
Pan-KDM4 Inhibitor TACH107 reduces proliferation and colony formation in PDAC cell lines *in vitro* and increases survival *in vivo*. **A,** MTT assay shows reduced proliferation in PDAC cell lines following TACH107 treatment, significance determined by one-way ANOVA. **B,** Reduced colony formation ability of human and mouse PDAC cell lines upon treatment with varying doses of TACH107 and (**C**) Quantification of the number of colonies, one-way ANOVA was used to assess significance. **D,** Survival plot showing B6 mice transplanted with PDAC cell line KPC-mT4 and treated with TACH107 survive longer than those treated with vehicle only (*n* = 3). **E,** Results from CETSA show increased heat stability of KDM4 proteins when treated with TACH107 vs. DMSO. **F,** Representative IF images showing increased H3K9me3 signal after TACH107 treatment compared with control cells. **G,** Normalized fluorescence intensity of H3K9me3 signal calculated by the corrected total cell fluorescence method using ImageJ, significance determined via unpaired Student *t* test. For all plots; * is *P* ≤ 0.05; ** is *P* ≤ 0.01; *** is *P* ≤ 0.001; and **** is *P* ≤ 0.0001. **H,** Representative micrographs showing H3K9me3 levels by IHC in PDAC tumors harvested on day 30 from mice injected with PDAC cell line KPC-mT4 demonstrate increased H3K9me3 mark in the TACH107 treated mice compared with vehicle control mice. Scale bar, 100 μm. **I,** Quantification of the area of positive staining in **H** using ImageJ color deconvolution plugin for IHC DAB staining, the difference between vehicle-treated and TACH107-treated samples is statistically significant.

In light of the observed upregulation of a related family member KDM4A in adapted *KDM4C*-null cells, we aimed to assess the effectiveness of an experimental pan-KDM4 small-molecule inhibitor in pancreatic cancer models *in vitro* and *in vivo*. We evaluated TACH107, an α-ketoglutarate competitive pan-KDM4 inhibitor, across three distinct PDAC cell lines. We treated two human PDAC cell lines AsPC1 and Pa03C and mouse PDAC cell line KPC-mT4 with varying doses of TACH107 and evaluated changes in viability and clonogenic potential in response to the drug. We observed a significant reduction in both cell viability and clonogenic potential *in vitro* across the three PDAC cell lines starting at submicromolar doses of TACH107 (Fig. 5A–C). Additionally, orthotopic transplantation of parental KPC-mT4 PDAC cells (with WT *KDM4C*) into the pancreas of B6 mice followed by treatment with TACH107 at a dose of 80 mg/kg/day resulted in a statistically significant improvement in survival compared with vehicle-treated controls (Fig. 5D). To verify the drug’s binding to its targets, we conducted a CETSA and observed that all KDM4 family members were stabilized against heat deactivation when treated with TACH107, which confirms that TACH107 binds to pan-KDM4 proteins (Fig. 5E). To validate that TACH107 binding leads to functional inactivation of KDM4 proteins, we carried out IF staining to measure changes in H3K9me3 levels with and without TACH107 in human and murine PDAC cells. We observed an increased signal intensity of H3K9me3 in treated cells compared with controls, indicating successful inactivation of KDM4 demethylase function (Fig. 5F and G). Similarly, the tumors harvested from TACH107 treated mice exhibited higher H3K9me3 levels on IHC compared with vehicle controls (Fig. 5H and I), whereas no consistent changes in KDM4C or KDM4A protein levels were observed (Supplementary Fig. S5A) In addition, HPNE cells exhibited minimal sensitivity to TACH107 compared with AsPC1 cells (Supplementary Fig. S5B), suggesting that the antiproliferative effect of pan-KDM4 inhibitor TACH107 is at least partially dependent on aberrant *KDM4C *expression. We conclude that small-molecule inhibition with the panKDM4 inhibitor TACH107 effectively slows pancreatic cancer tumor growth with demonstrable on-target effects.

### Proximity labeling identifies a novel KDM4C–SIRT1–DUSP2 axis that regulates ERK activity in PDAC cells

**Figure 6.**
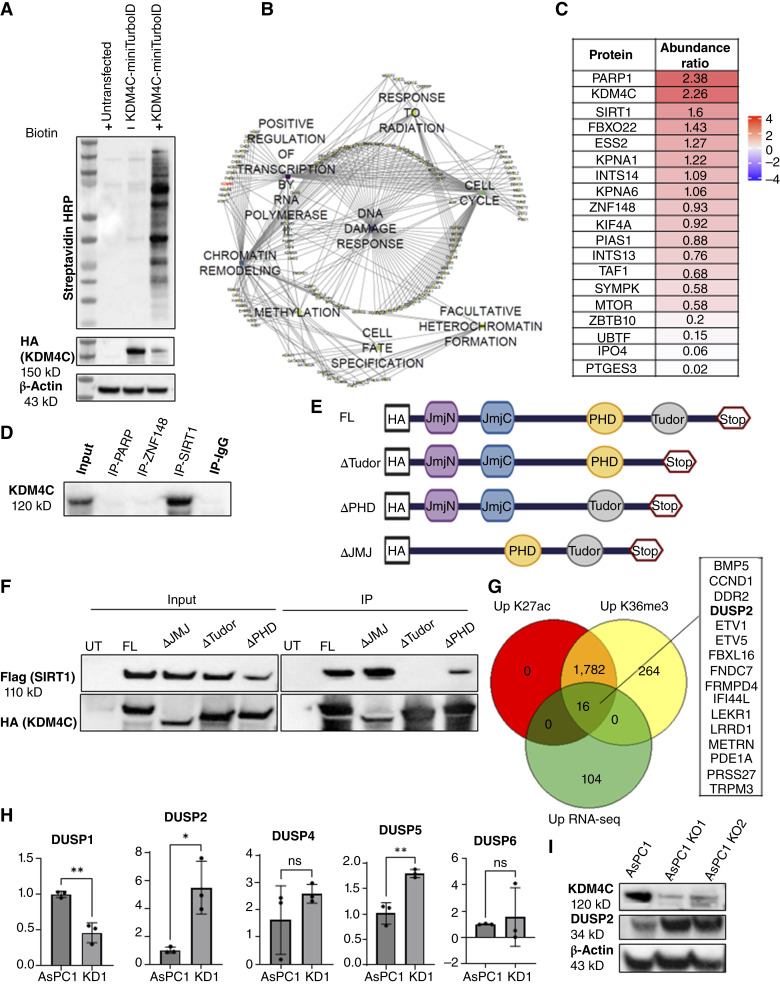
Proximity labeling identifies histone acetylase SIRT1 as a direct KDM4C nuclear interactor and a novel KDM4C–SIRT1–DUSP2 axis regulating ERK activity in PDAC. **A,** Validating biotinylation activity by KDM4C-miniTurboID. AsPC1 cells were transfected with KDM4C-miniTurboID and then treated with 50 μmol/L biotin 24 hours after transfection for 30 minutes. Cells were then lysed and the whole lysates probed for biotin by streptavidin–HRP along with “untransfected” and “KDM4C-miniTurboID without biotin” as controls. **B,** Cytoscape analysis of the biotinylated nuclear proteins by KDM4C-miniTurboID, with annotated biological processes. **C,** List of high-confidence nuclear KDM4C interactors based on proximity labeling experiment. The values shown are the abundance ratio between “KDM4C-miniTurboID + biotin” over “KDM4C-miniTurboID without biotin”. **D,** KDM4C co-immunoprecipitation confirms binding to SIRT1 in PDAC cells but not to PARP1 or ZNF148. IgG isotype antibody was used as a control for nonspecific binding. **E,** Graphical illustration of KDM4C domain-specific deletion constructs: FL = full-length KDM4C construct, ΔTudor = Tudor domain–deleted KDM4C, ΔPHD = PHD-deleted KDM4C, and ΔJMJ = JMJ domain–deleted KDM4C construct. All constructs are HA-tagged for immunoprecipitation. **F,** The Tudor domain is responsible for KDM4C binding to SIRT1. HEK293 cells were cotransfected with Flag-tagged SIRT1 construct and either FL KDM4C or a KDM4C domain–deleted construct, and then the cells were lysed, and the HA-enriched eluates for respective samples were probed for SIRT1 using anti-Flag. All constructs could pull down SIRT1 except ΔTudor. **G,** Venn diagram illustrating high degree of correlation between the genes with increased H3K36me3 and H3K27Ac peaks at the promoter region in *KDM4C* KO cells. Integrating ChIP-seq with the RNA-seq data results in a list of 16 genes, including the protein phosphatase *DUSP2*, which targets ERK phosphorylation. **H,** RT-qPCR of *DUSP* 1, 2, 4, 5, and 6 in AsPC1 control cells and *KDM4C* KO shows *DUSP2* has the highest FC increase in mRNA expression, *, *P* ≤ 0.05; **, *P* ≤ 0.01, via unpaired *t* test. **I,** Western blot shows increased DUSP2 protein levels in *KDM4C*-null clones.

As KDM4C does not directly regulate phosphorylation levels in proteins, we sought to identify a candidate mediator of the observed effects on ERK activation upon acute KDM4C loss. To identify putative binding partners of KDM4C, we performed proximity labeling using TurboID followed by mass spectrometry analysis. In this technique, TurboID biotin ligase uses ATP to biotinylate proteins in proximity to bait protein which can then be enriched using streptavidin beads and analyzed (24, 30). As KDM4C principally functions in the nucleus, we created an HA-tagged KDM4C-miniTurboID fusion construct with a nuclear localization signal and confirmed the construct expression and localization when transfected into PDAC cell lines using IF and Western blotting (Supplementary Fig. S6A–S6C). We used AsPC1 cells transfected with KDM4C-miniTurboID and incubated with biotin to identify KDM4C-interacting proteins in the nucleus (Fig. 6A). The interactome map shows that KDM4C interacts with proteins involved in known KDM4C functions, such as histone demethylation, chromatin remodeling, and regulating transcription. In addition, several novel biological functions were identified from the network like DNA damage response and cell cycle (Fig. 6B). Top interacting proteins with KDM4C included the DNA repair enzyme PARP1 and the class III HDAC SIRT1, neither of which have been previously described as direct KDM4C interactors (Fig. 6C). We chose three of the top identified hits, PARP1, SIRT1, and ZNF148, to validate their binding with KDM4C using co-immunoprecipitation. The results confirm KDM4C binding to SIRT1 but not PARP1 or ZNF148 (Fig. 6D). Furthermore, to determine whether KDM4C binding to SIRT1 is related to its demethylase functionality, we created domain-specific deletion constructs for each of the three KDM4C protein domains, namely the Tudor and PHD reader domains and the JMJ demethylation catalytic domain (Fig. 6E). Each domain-deleted construct has an HA-tag to allow for specific pull down. We cotransfected HEK293 cells with Flag-SIRT1 construct and either full-length HA-tagged KDM4C construct (FL) or one of the domain deletion constructs: ΔTudor, ΔPHD, or ΔJMJ. Deletion of the Tudor domain of KDM4C resulted in loss of binding to SIRT1, suggesting that the Tudor domain is required for interaction between the two proteins (Fig. 6F).

We hypothesized that KDM4C and SIRT1 could be repressing downstream genes in PDAC that regulate ERK activity, by demethylating the H3K36me3 mark associated with active gene transcription and deacetylating the H3K27Ac mark associated with active enhancer regions, respectively. To this end, we performed H3K36me3 and H3K27Ac ChIP-seq on parental AsPC1 and *KDM4C* KO cells and interrogated genes that had both increased H3K36me3 and H3K27Ac near the promoter region in the *KDM4C*-null cells (Heatmap from ChIP-Seq data in Supplementary Fig. S6D). We then integrated the results from the ChIP-seq analysis with the RNA-seq analysis and compiled a list of genes that had both upregulated H3K36me3 and H3K27Ac marks, and the corresponding transcripts were significantly overexpressed in the RNA-seq dataset from AsPC1 KO1. Interestingly, the genes that exhibited upregulated H3K36me3 peaks largely correlated with those with upregulated H3K27Ac peaks (Fig. 6G). A list of 16 transcripts fit this stringent filtering criteria, including dual-specificity protein phosphatase 2 or *DUSP2*. *DUSP2* encodes for a phosphatase that negatively regulates ERK protein activity by dephosphorylation (31). These data suggest that DUSP2 could be a target of KDM4C and SIRT1 repression, resulting in the activation of ERK signaling. As there are several DUSP enzymes involved in ERK protein inactivation, we asked whether KDM4C specifically regulates *DUSP2*. We screened levels of *DUSP 1*, *2*, *4*, *5*, and *6*, which are all known modulators of ERK signaling, using quantitative RT-PCR in AsPC1 and early-passage KDM4C KD cells. We found that *DUSP2* had the highest FC increase in mRNA expression (Fig. 6H). Additionally, *DUSP5* was significantly increased in the knockdown sample, although the fold increase was not as high as *DUSP2*, and *DUSP5* was not differentially expressed in the RNA-seq data (Log_2_ FC = 0.237, adjusted *P* value = 0.457). Western blot analysis confirmed increased DUSP2 protein levels in the absence of KDM4C in AsPC1 cells (Fig. 6I), whereas knocking down *DUSP2* using siRNA could partially restore pERK levels in the absence of KDM4C (Supplementary Fig. S6E). Our data indicate an interaction between nuclear KDM4C and SIRT1 contributes to epigenetic repression of downstream targets such as the phosphatase DUSP2. This repression of *DUSP2* is at least one of the factors associated with increased ERK phosphorylation in PDAC, representing a novel approach to targeting this pivotal oncogenic effector in the setting of mutant RAS.

## Discussion

In this study, we establish that KDM4C is overexpressed in PDAC compared with non-neoplastic pancreas and that the protein functionally contributes to PDAC cell proliferation and growth. This is in line with current knowledge on KDM4C overexpression in other cancer types (10, 11, 32–34). Studies on KDM4C in different cancer types have revealed pleiotropic effector signaling axes downstream of KDM4C, which emphasizes context and cell type dependency. In prostate cancer, KDM4C regulates androgen receptor signaling, amplifying androgen-dependent proliferation in prostate tumor cells through trimethyl H3K9 demethylation (9). In lung cancer, KDM4C promoted metastasis and epithelial–mesenchymal transition via CUL4A (12), whereas it enhanced sphere formation by activating the notch ligand JAG1 via the β-catenin pathway in colorectal cancer (33, 35). Our data suggest that KDM4C’s oncogenic role in pancreatic cancer is executed through at least two mechanisms: cancer cell–intrinsic sustenance of ERK activation and non–cell-autonomous immune suppression. We uncovered a robust correlation between *KDM4C* expression and ERK activation through extensive epigenomic, transcriptomic, and proteomic profiling, which was validated in human and murine PDAC models. Knocking out *KDM4C* results in reduced phosphorylation of ERK1/2 and a global transcriptomic signature similar to ERK inhibition. We have identified that SIRT1, a class III HDAC, is a nuclear binding partner of KDM4C. SIRT1 binds to KDM4C via the reader Tudor domain independently of the JMJ catalytic domain of KDM4C and represses the transcription of downstream genes by removing the active H3K27Ac and H3K36me3 marks, respectively. Transcriptomic and epigenomic data imply that KDM4C and SIRT1 could epigenetically repress *DUSP2* expression, which is likely a major contributor to ERK regulation by KDM4C although it may not be the only mediator. Under prolonged loss of KDM4C function, PDAC cells restore active ERK levels and rescue the *in vitro* growth phenotype. This behavior is reminiscent of how patients with cancer develop resistance to KRAS inhibitors by activating orthogonal bypass mechanisms, including upregulation of upstream receptor tyrosine kinases and growth factor ligands (36). Overall, our findings point to a potential KDM4C–SIRT1–DUSP2 axis that may contribute to ERK activation in KRAS-mutant PDAC. Although this proposed regulatory pathway offers a novel framework for understanding epigenetic modulation of KRAS signaling, further functional interrogation is necessary to establish its mechanistic relevance and therapeutic implications, particularly in the context of emerging pharmacologic inhibitors targeting KRAS-driven pathways.

Interestingly, although the adapted *KDM4C*-null cells demonstrate a rescue of their *in vitro* growth phenotypes and *in vivo* growth characteristics in immune-compromised mice comparable with that of *KDM4C* WT parental cells, there is persistent attenuation of growth *in vivo* in immune-competent settings, suggesting a KDM4C-specific modulatory effect on the immune system that is not rescued by restoring ERK signaling. Immunophenotypic analysis demonstrated significant reduction in tumor-promoting immune cells, including monocytic MDSC, and an influx of tumor-infiltrating B cells and CD8^+^ T cells that lead to enhanced immune surveillance. The mediators of these “KDM4C-specific” effectors on the immune system remain under examination but could serve as a basis for combining KDM4C inhibition with immunotherapeutic approaches.

Recently, a clinical grade pan-KDM4 inhibitor TACH101 has been developed, which has demonstrated efficacy in preclinical models of multiple solid cancers and entered phase 1 clinical trials (21). We tested a cognate tool compound TACH107 and found it is effective in attenuating the growth of PDAC models *in vitro* and *in vivo*. The compound also confirmed on-target biological effects such as enhanced H3K9 levels in cells exposed to the compound and evidence of binding to multiple KDM proteins on CETSA. The availability of a panKDM4 inhibitor in some respects mirrors the advantages of a panKRAS antagonist versus an allele-specific antagonist in that TACH107 (or the clinical grade TACH101) can circumvent the cell-intrinsic adaptive mechanism that restores ERK activation. At the same time, like panKRAS antagonists, a panKDM4 inhibitor likely also leads to greater on-target, off-tumor toxicities because of the requirement of various KDM4 proteins in homeostasis. As the clinical trial data mature, the competing effects of tumor efficacy versus adverse events will become more evident.

The shortcomings of our data include the absence of an autochthonous model to fully explore the role of *KDM4C* deletion from early cancer within a complex host microenvironment. Our allograft experiments demonstrate that much like other epigenetic drivers, KDM4C has pleiotropic effects, including ones that cannot be compensated by related KDM4 family members. Genetic models would help elucidate these pleiotropic effects with greater nuance. We also have not explored the effects of *KDM4A* deletion in the setting of compensation to determine whether it is the main compensatory mechanism or whether there exists a “whack-a-mole” phenomenon within the overall KDM4 family for other members stepping in to restore pivotal ERK signaling. Finally, although we describe ERK regulation as a novel and indirect KDM4C function in KRAS-mutant PDAC, the role of this axis in other KRAS-mutant cancers like colorectal and lung cancers is uncertain. A tissue agnostic effect would substantially enhance the translational potential of our findings.

## Supplementary Material

Supplementary Table 1 CRISPR sgRNA sequences: sequence information for sgRNAs used to knockout *KDM4C* in human and mouse PDAC cell lines.

Supplementary Table 2 PCR primer sequences: Sequence information for forward and reverse primers used to detect mRNA levels of *DUSP1*, *DUSP2*, *DUSP4*, *DUSP5*, and *DUSP6*.

Supplementary Table 3 *KDM4C* shRNA sequences: Oligo ID and sequence information for the shRNA used to knockdown *KDM4C*.

Supplementary Table 4 CyTOF Antibody Panel: Information about antibodies used in CyTOF experiment, their target, source, clones, and cat. no.

Supplementary Figure 1 Related to Figure 2: effect of *KDM4C *KO on the proliferation of HPNE cells.

Supplementary Figure 2 Related to Figure 3: PCA and heatmap of DE genes from RNAseq data and the effect of ERK inhibition on *KDM4C* KO vs WT PDAC cells.

Supplementary Figure 3 Related to Figure 4: Heatmap from RPPA data on KPC-mT4 cells and adapted *KDM4C* KO clones.

Supplementary Figure 4 Related to Figure 4: KDM4C depletion increases immune surveillance in vivo.

Supplementary Figure 5 Related to Figure 5: Effect of TACH107 on KDM4A and KDM4C levels, and on HPNE viability.

Supplementary Figure 6 Related to Figure 6: KDM4C-miniTurboID validation, Heatmaps for H3K36me3 and H3K27Ac ChIPseq in *KDM4C* KO vs WT AsPC1, and *DUSP2 *knockdown western panel.

Supplementary Figure 7 Full blots: Uncropped blot images for western blot panels shown in the main and supplementary figures followed by Imagetwin reports with comments on detected issues.

## Data Availability

The datasets generated and analyzed during this study have been deposited in the Gene Expression Omnibus under accession number GSE300200. All other data are in the main manuscript, supplemental files, or are available upon request from the corresponding author.
