# Supplementary material for: The Lysine Demethylase KDM4C Is an Oncogenic Driver and Regulates ERK Activity in KRAS-Mutant Pancreatic Ductal Adenocarcinoma
Source: Cancer Res Commun. 2026 Jan 30;6(1):245–59. doi: 10.1158/2767-9764.CRC-25-0278 (PMC12856980; doi:10.1158/2767-9764.CRC-25-0278)
Supplement: Supplementary Table 1 — CRISPR sgRNA sequences: sequence information for sgRNAs used to knockout KDM4C in human and mouse PDAC cell lines. [file crc-25-0278_supplementary_table_1_suppst1.docx]

**Supplementary Table 1: CRISPR sgRNA sequences**

| **KDM4C sgRNA** | **Sequence 5’-3’** |
| --- | --- |
| SgKDM4C-1 | TAGTGAATCGAACTTCTGG |
| SgKDM4C-2 | AGAGGTCCATGTCTTCGGT |
| SgKdm4c-1 | ATATCATCATAGCACTGTCT |
| SgKdm4c-2 | GCAGATGGTCACAGGGCAGT |
