# Supplementary material for: The Lysine Demethylase KDM4C Is an Oncogenic Driver and Regulates ERK Activity in KRAS-Mutant Pancreatic Ductal Adenocarcinoma
Source: Cancer Res Commun. 2026 Jan 30;6(1):245–59. doi: 10.1158/2767-9764.CRC-25-0278 (PMC12856980; doi:10.1158/2767-9764.CRC-25-0278)
Supplement: Supplementary Table 2 — PCR primer sequences: Sequence information for forward and reverse primers used to detect mRNA levels of DUSP1, DUSP2, DUSP4, DUSP5, and DUSP6. [file crc-25-0278_supplementary_table_2_suppst2.docx]

**Supplementary Table 2: PCR primer sequences**

| **Primer** | **Sequence 5’-3’** |
| --- | --- |
| Human *DUSP1* forward | CCTCTGGGTTTCTAAGCAGTTAT |
| human *DUSP1* reverse | GTCAGATGGACTTGATGTACCC |
| human *DUSP2* forward | CTCCTCCATCTTGCTCAACTC |
| human *DUSP2* reverse | TGTAGCCATCTGCATCCTTG |
| human *DUSP4* forward | GCCTATGTCCTGATCCATGTG |
| human *DUSP4* reverse | CCAGCTCATGTGGCTCTTT |
| human *DUSP5* forward | CTGAGTGTTGCGTGGATGTA |
| human *DUSP5* reverse | CTGGCCTGTAGCTGACATTTA |
| human *DUSP6* forward | CAACCTCTATTCCCAGCTTTCC |
| human *DUSP6* reverse | CCATGTTCCAGGCAGTGTTAT |
