# Supplementary material for: The Lysine Demethylase KDM4C Is an Oncogenic Driver and Regulates ERK Activity in KRAS-Mutant Pancreatic Ductal Adenocarcinoma
Source: Cancer Res Commun. 2026 Jan 30;6(1):245–59. doi: 10.1158/2767-9764.CRC-25-0278 (PMC12856980; doi:10.1158/2767-9764.CRC-25-0278)
Supplement: Supplementary Table 3 — KDM4C shRNA sequences: Oligo ID and sequence information for the shRNA used to knockdown KDM4C. [file crc-25-0278_supplementary_table_3_suppst3.docx]

**Supplementary Table 3: shRNA sequences**

| **KDM4C shRNA Oligo ID** | **Sequence 5’-3’** |
| --- | --- |
| V3LHS_337727 | TAGTGAATCGAACTTCTGG |
| V3LHS_337731 | AGAGGTCCATGTCTTCGGT |
