# Supplementary material for: The Lysine Demethylase KDM4C Is an Oncogenic Driver and Regulates ERK Activity in KRAS-Mutant Pancreatic Ductal Adenocarcinoma
Source: Cancer Res Commun. 2026 Jan 30;6(1):245–59. doi: 10.1158/2767-9764.CRC-25-0278 (PMC12856980; doi:10.1158/2767-9764.CRC-25-0278)
Supplement: Supplementary Table 4 — CyTOF Antibody Panel: Information about antibodies used in CyTOF experiment, their target, source, clones, and cat. no. [file crc-25-0278_supplementary_table_4_suppst4.docx]

**Supplementary Table 4: CyTOF antibody panel**

|  |  |  |  |  |
| --- | --- | --- | --- | --- |
| **Tagged antibody description** | **Target** | **Clone** | **Source** | **Cat. No.** |
| CD11c(Ms) 142Nd | CD11c | N418 | DVS-Fluidigm | 3142003B |
| Ly-6C 89Y | Ly-6C | HK1.4 | BioLegend | 128002 |
| CD3(Ms) 151Eu | CD3 | 17A2 | Tonbo | 70-0032-U500 |
| F4/80 159Tb (MDA) | F4/80 | BM8 | BioLegend | 123102 |
| CD86,B7-2 | CD86,B7-3 | A17199A | BioLegend | 159202 |
| NK1.1 170Er (MDA) | NK1.1 | PK136 | BioLegend | 108702 |
| Siglec-F 172Yb | Siglec-F | E50-2440 | BD | 552125 |
| CD279(Ms) 161Dy (29F.1A12) | CD279, PD-1 | 29F.1A12 | BioLegend | 135202 |
| CD45(Ms) 152Sm | CD45 | 30-F11 | Tonbo | 70-0451-U100 |
| FceR1a 156Gd | FceR1a | MAR-1 | BioLegend | 134321 |
| CD11b 139La | CD11b | M1/70 | BioLegend | 101249 |
| CD44 111Cd | CD44 | IM7 | Tonbo | 70-0441-U100 |
| CD163 | CD163 | S15049F | BioLegend | 156702 |
| CD8a | CD8a | 53-6.7 | BioLegend | 100755 |
| CD19(Ms) 149Sm (IMC) | CD19 | 1D3 | Tonbo | 70-0193-U100 |
| CD69(Ms) 166Er | CD69 | H1.2F3 | BioLegend | 104533 |
| Ly-6G 141Pr | Ly-6G | 1A8 | DVS-Fluidigm | 3141008B |
| CD4(Ms) 145Nd (MDA) | CD4(Ms) | RM4-5 | BioLegend | 100506 |
| CD62L(Ms) 164Dy (MDA) | CD62L | MEL-14 | Tonbo | 70-0621-U100 |
| I-A/I-E(Ms) 174Yb | I-A/I-E, MHC-II | M5/114.15.2 | DVS-Fluidigm | 3174003B |
| CD138 | CD138 | 281-2 | BioLegend | 142502 |
| CD45 106 Cd | CD45 | 30-F11 | DVS-Fluidigm | 92J006106 |
| CD45 110 Cd | CD45 | 30-F11 | DVS-Fluidigm | 92J006110 |
| CD45 112 Cd | CD45 | 30-F11 | DVS-Fluidigm | 92J006112 |
| CD45 113 Cd | CD45 | 30-F11 | DVS-Fluidigm | 92J006113 |
| CD45 114 Cd | CD45 | 30-F11 | DVS-Fluidigm | 92J006114 |
| CD45 116 Cd | CD45 | 30-F11 | DVS-Fluidigm | 92J006116 |
