# Supplementary figures and images for: The Lysine Demethylase KDM4C Is an Oncogenic Driver and Regulates ERK Activity in KRAS-Mutant Pancreatic Ductal Adenocarcinoma
Source: Cancer Res Commun. 2026 Jan 30;6(1):245–59. doi: 10.1158/2767-9764.CRC-25-0278 (PMC12856980; doi:10.1158/2767-9764.CRC-25-0278)

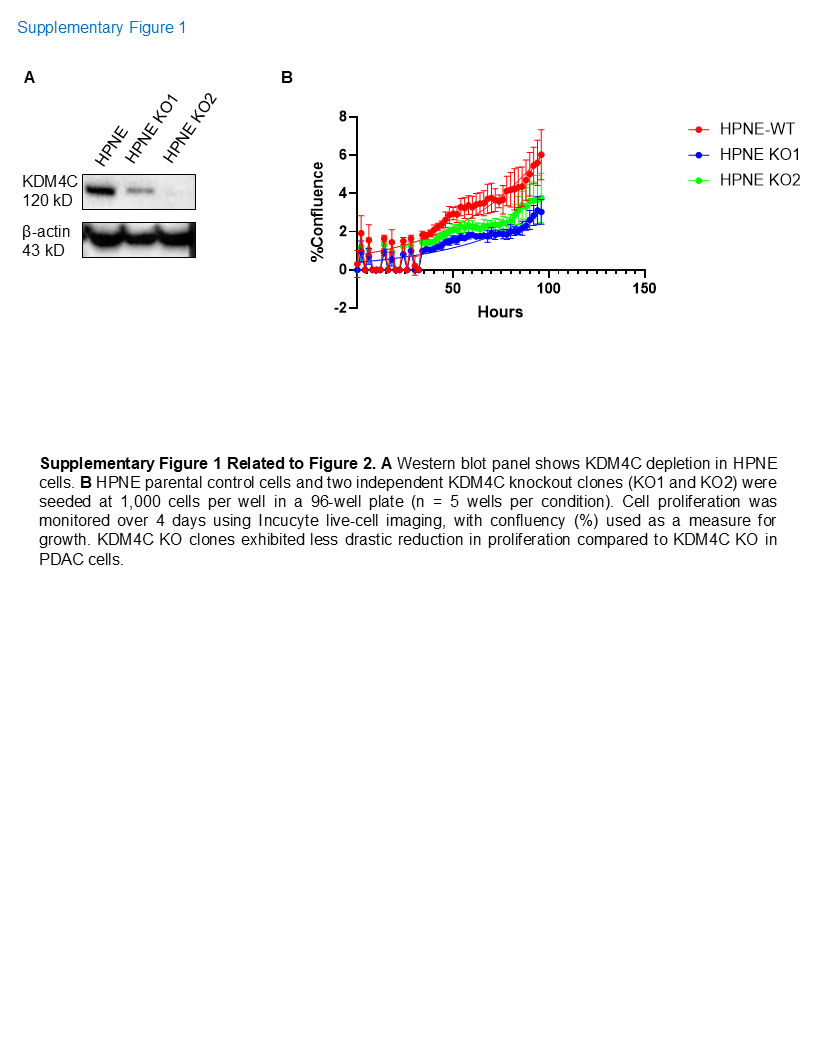

Supplement: Supplementary Figure 1 — Related to Figure 2: effect of KDM4C KO on the proliferation of HPNE cells. [file crc-25-0278_supplementary_figure_1_suppsf1.png]

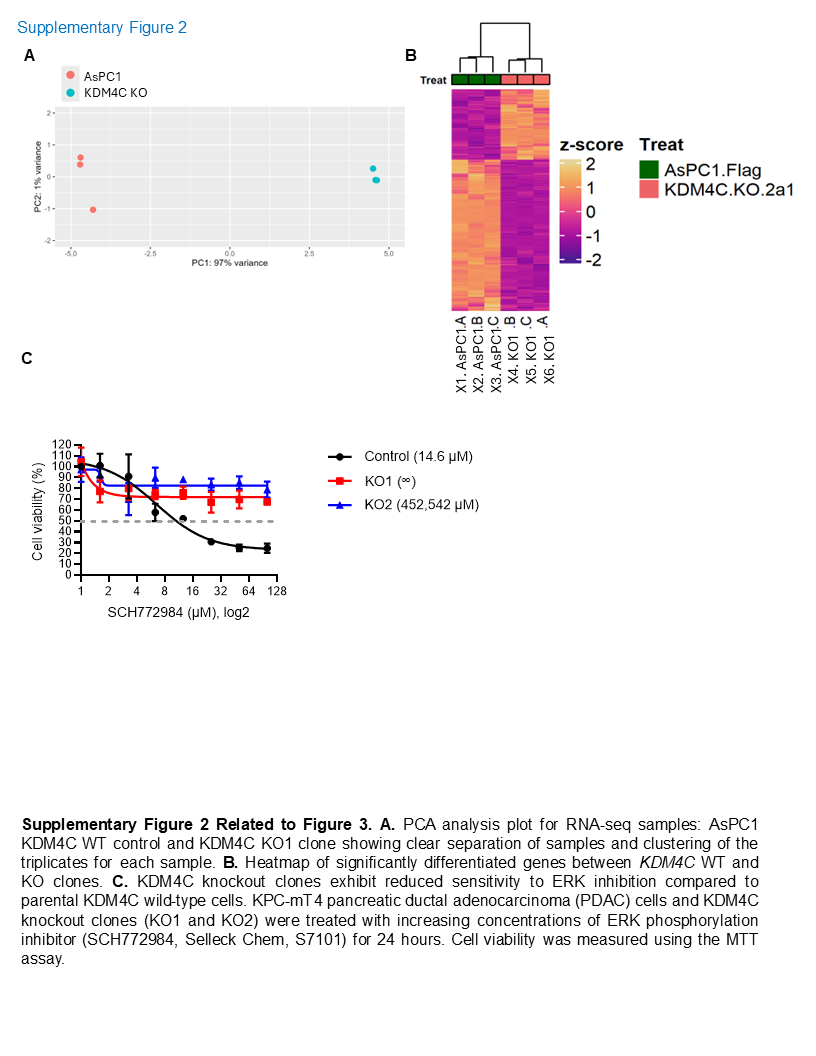

Supplement: Supplementary Figure 2 — Related to Figure 3: PCA and heatmap of DE genes from RNAseq data and the effect of ERK inhibition on KDM4C KO vs WT PDAC cells. [file crc-25-0278_supplementary_figure_2_suppsf2.png]

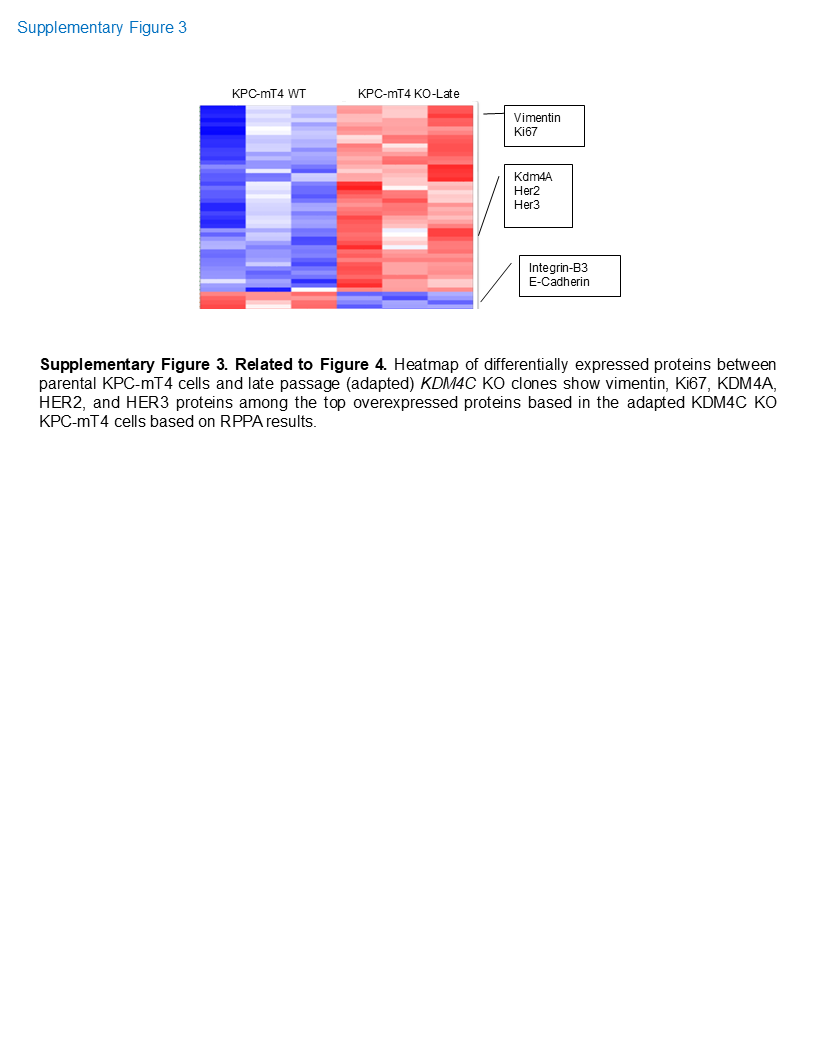

Supplement: Supplementary Figure 3 — Related to Figure 4: Heatmap from RPPA data on KPC-mT4 cells and adapted KDM4C KO clones. [file crc-25-0278_supplementary_figure_3_suppsf3.png]

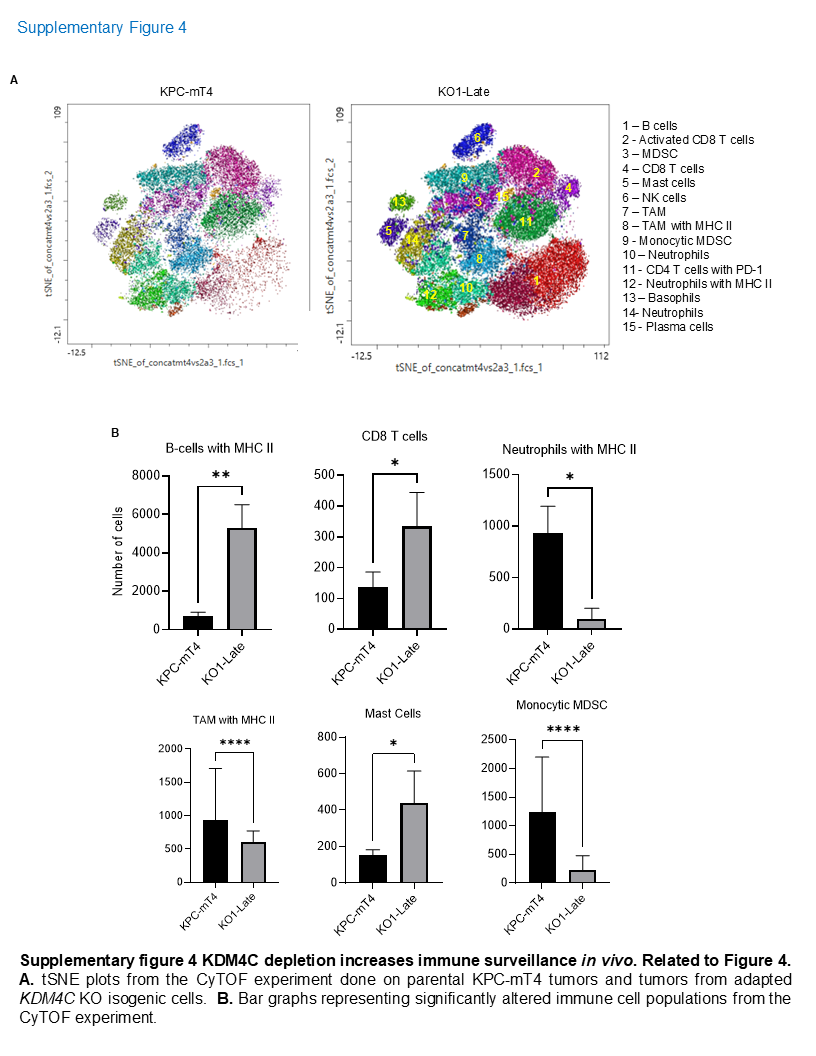

Supplement: Supplementary Figure 4 — Related to Figure 4: KDM4C depletion increases immune surveillance in vivo. [file crc-25-0278_supplementary_figure_4_suppsf4.png]

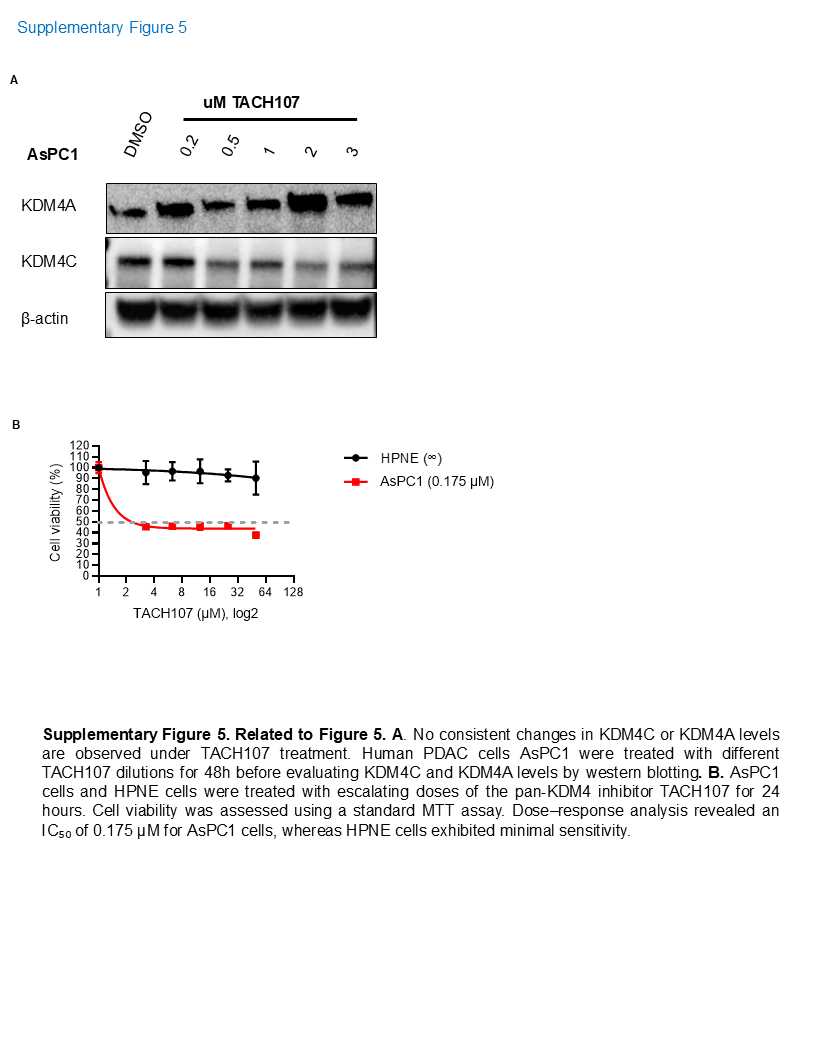

Supplement: Supplementary Figure 5 — Related to Figure 5: Effect of TACH107 on KDM4A and KDM4C levels, and on HPNE viability. [file crc-25-0278_supplementary_figure_5_suppsf5.png]

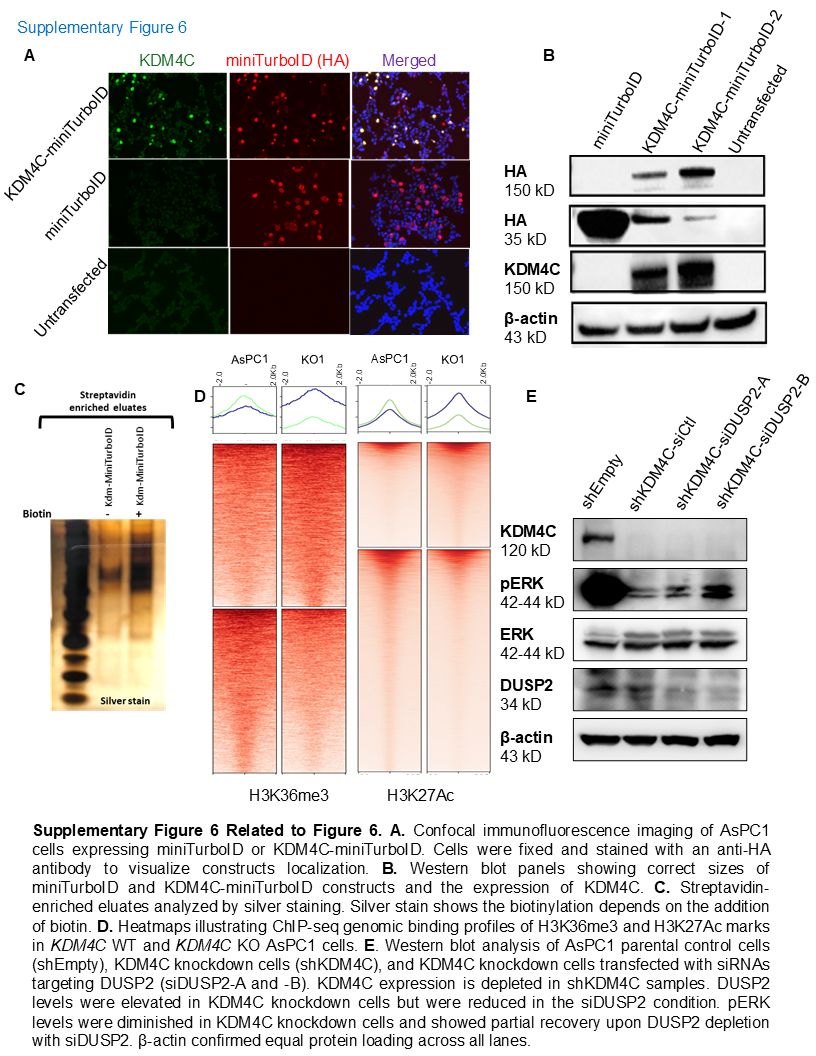

Supplement: Supplementary Figure 6 — Related to Figure 6: KDM4C-miniTurboID validation, Heatmaps for H3K36me3 and H3K27Ac ChIPseq in KDM4C KO vs WT AsPC1, and DUSP2 knockdown western panel. [file crc-25-0278_supplementary_figure_6_suppsf6.png]
