# Supplementary material for: The Lysine Demethylase KDM4C Is an Oncogenic Driver and Regulates ERK Activity in KRAS-Mutant Pancreatic Ductal Adenocarcinoma
Source: Cancer Res Commun. 2026 Jan 30;6(1):245–59. doi: 10.1158/2767-9764.CRC-25-0278 (PMC12856980; doi:10.1158/2767-9764.CRC-25-0278)
Supplement: Supplementary Figure 7 — Full blots: Uncropped blot images for western blot panels shown in the main and supplementary figures followed by Imagetwin reports with comments on detected issues. [file crc-25-0278_supplementary_figure_7_suppsf7.pdf]

Supplementary Figure 7: Full Blots followed by Imagetwin report

Figure 1A

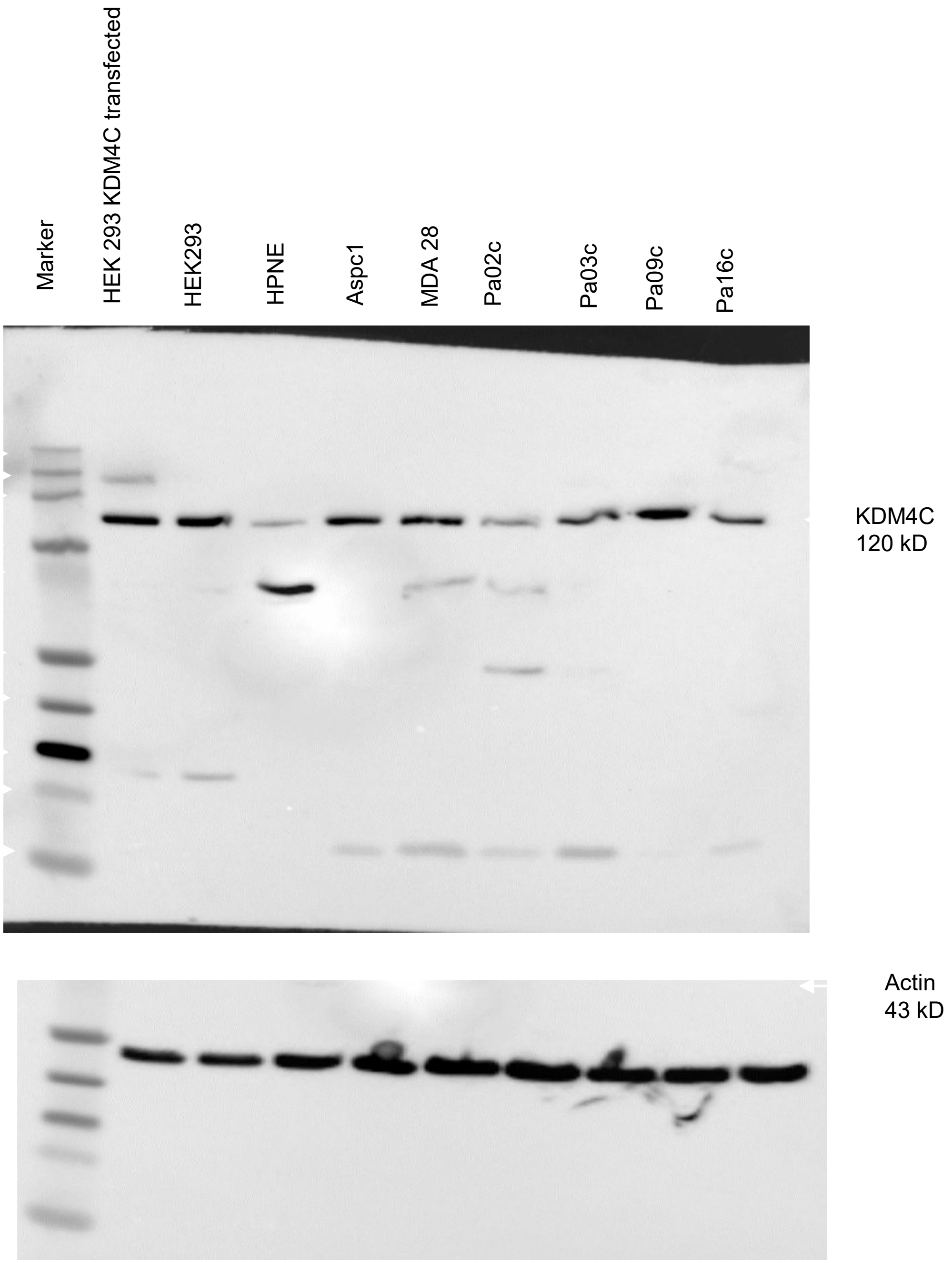

Figure 2A

KDM4C
120 kD

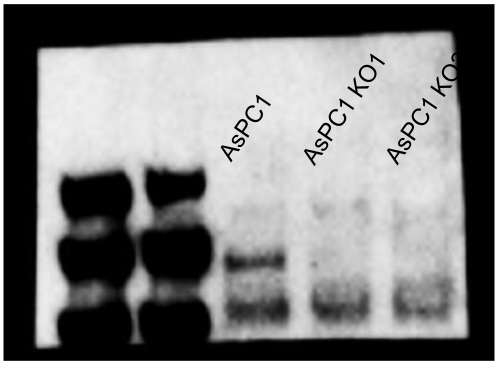


β-actin
43 kD

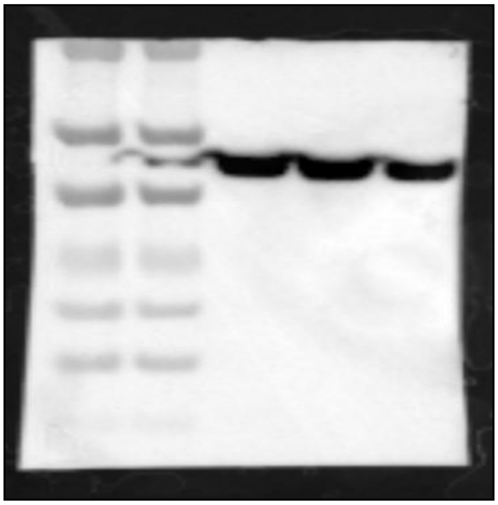

KDM4C
120 kD

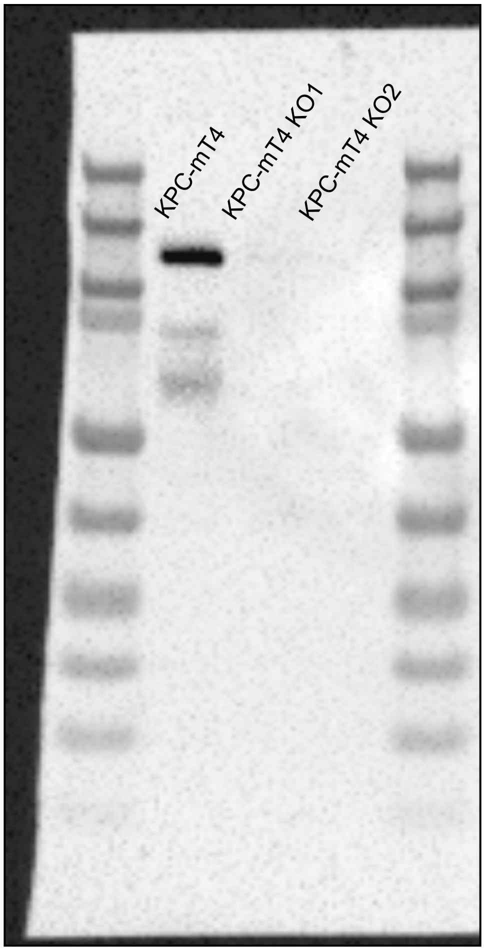


β-actin
43 kD

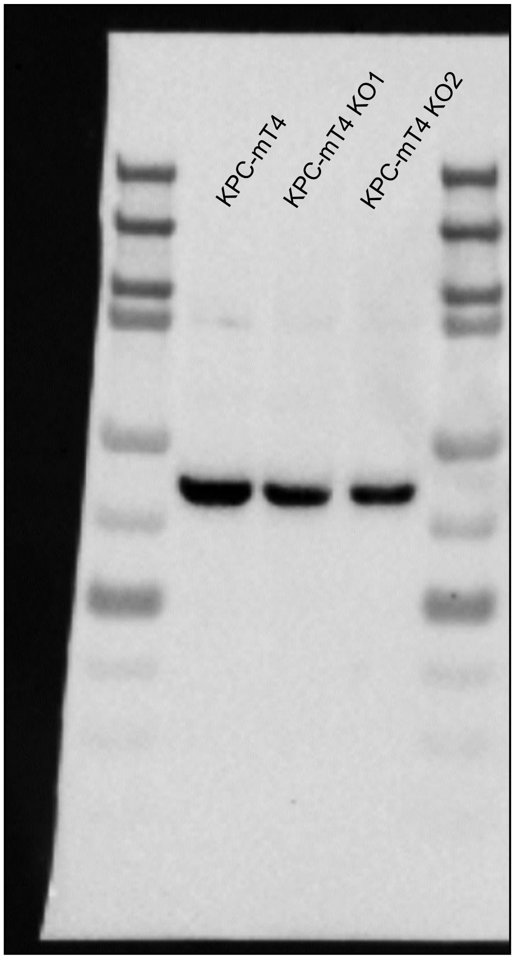

Figure 3D:
AsPC1 KO1 KO2

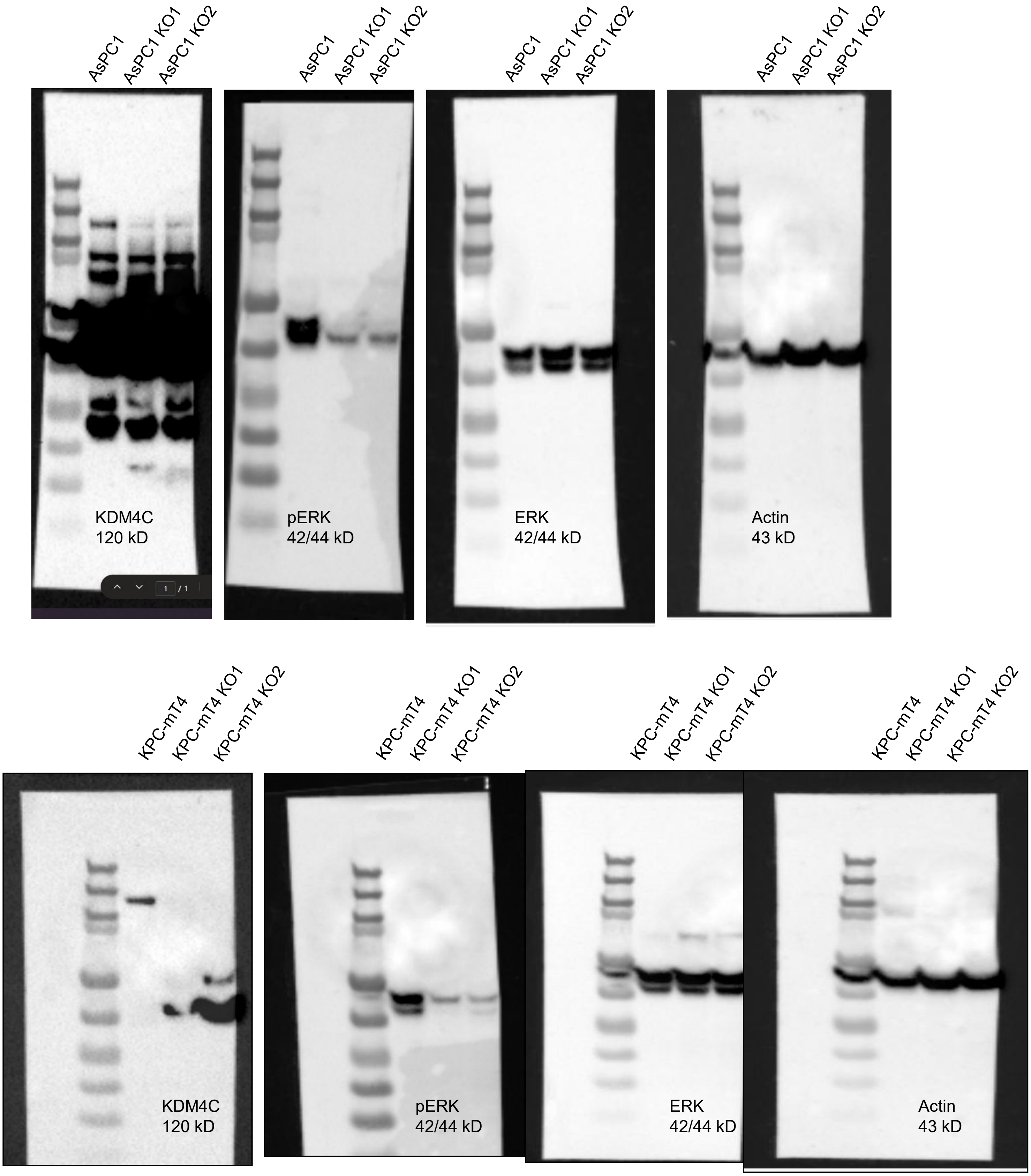

Figure 4A

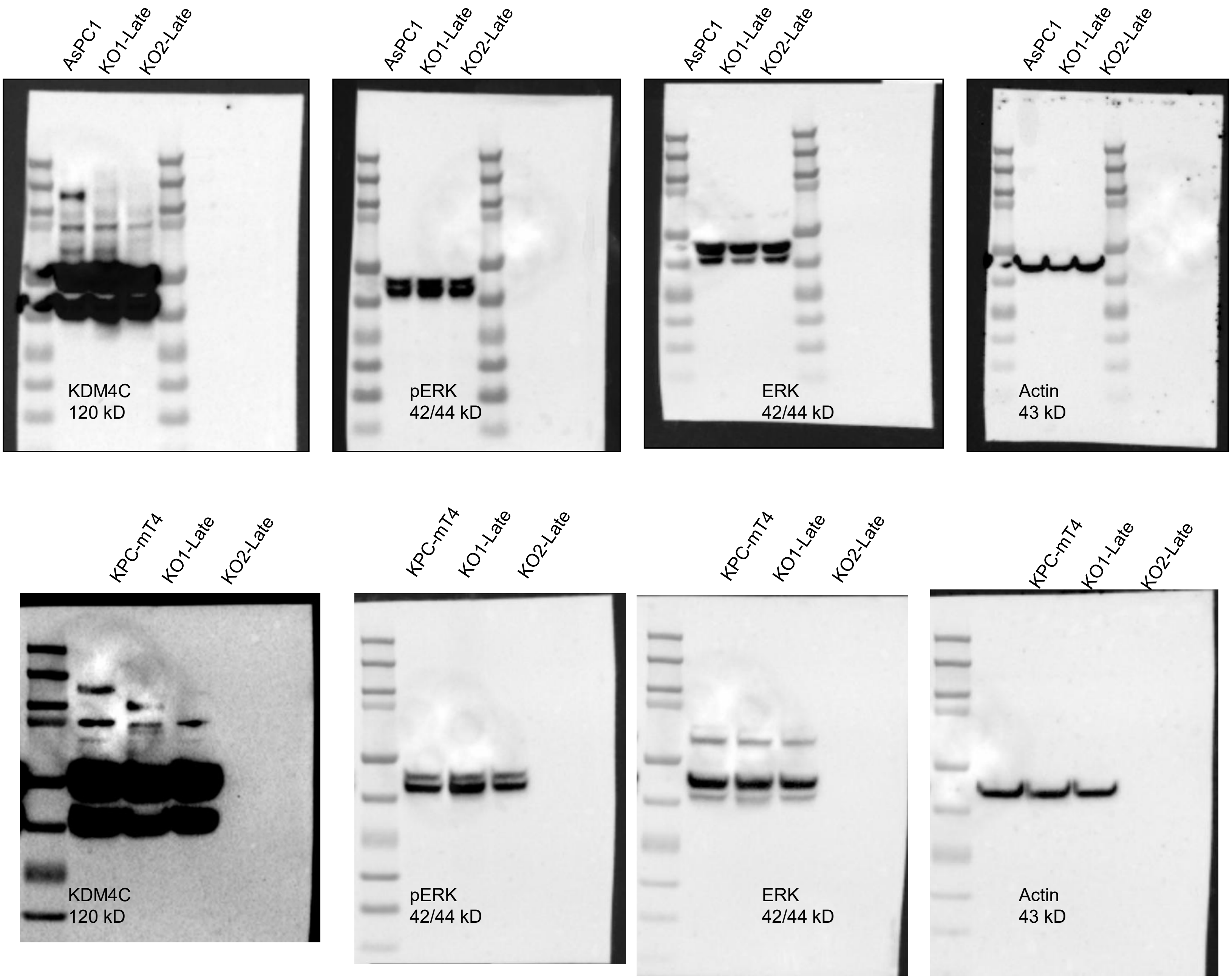
AsPC1
KO1-Late
KO2-Late
KDM4C
120 kD
pERK
42/44 kD
ERK
42/44 kD
Actin
43 kD
KPC-mT4
KO1-Late
KO2-Late
KDM4C
120 kD
pERK
42/44 kD
ERK
42/44 kD
Actin
43 kD

Figure 4E

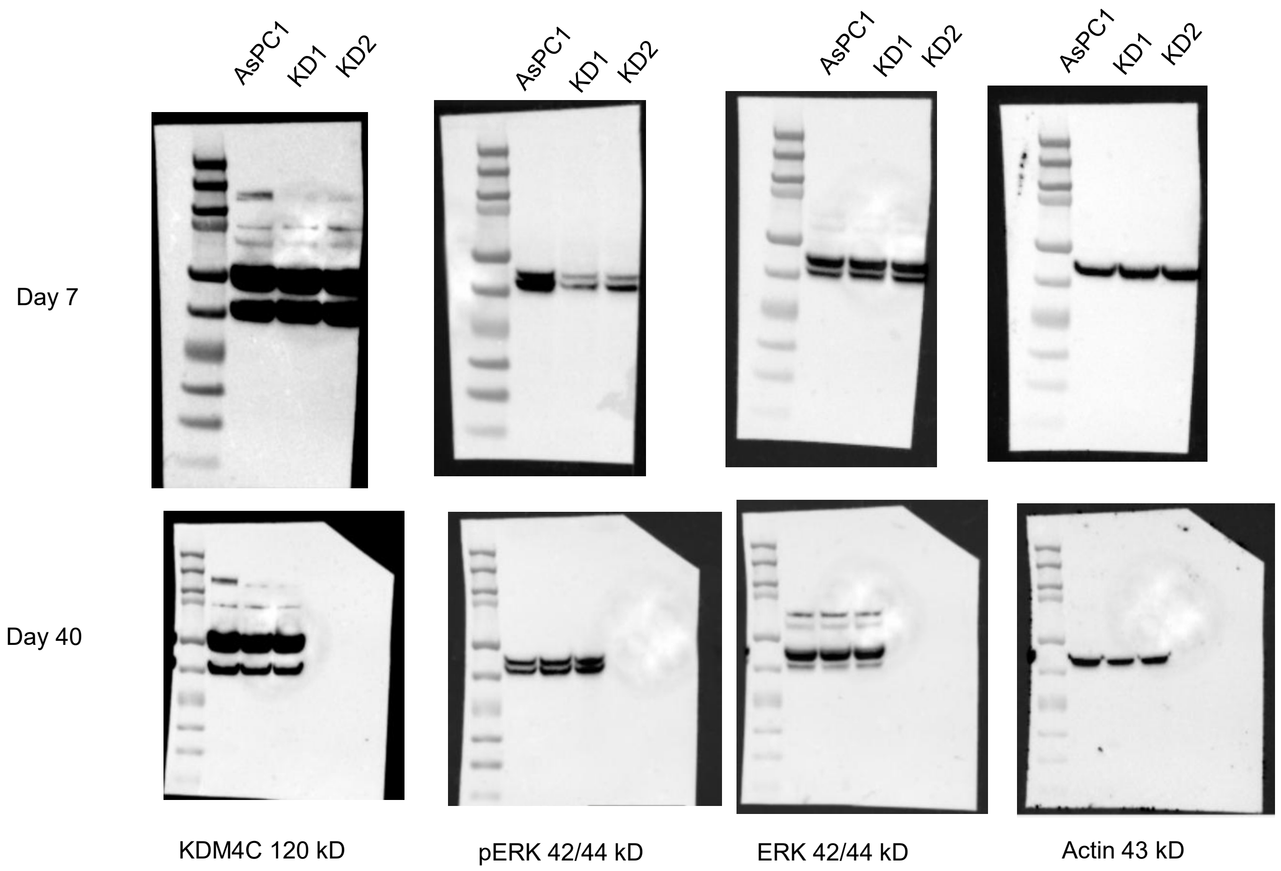
AsPC1
KD1
KD2
Day 7
Day 40
KDM4C 120 kD
pERK 42/44 kD
ERK 42/44 kD
Actin 43 kD

Figure 4F

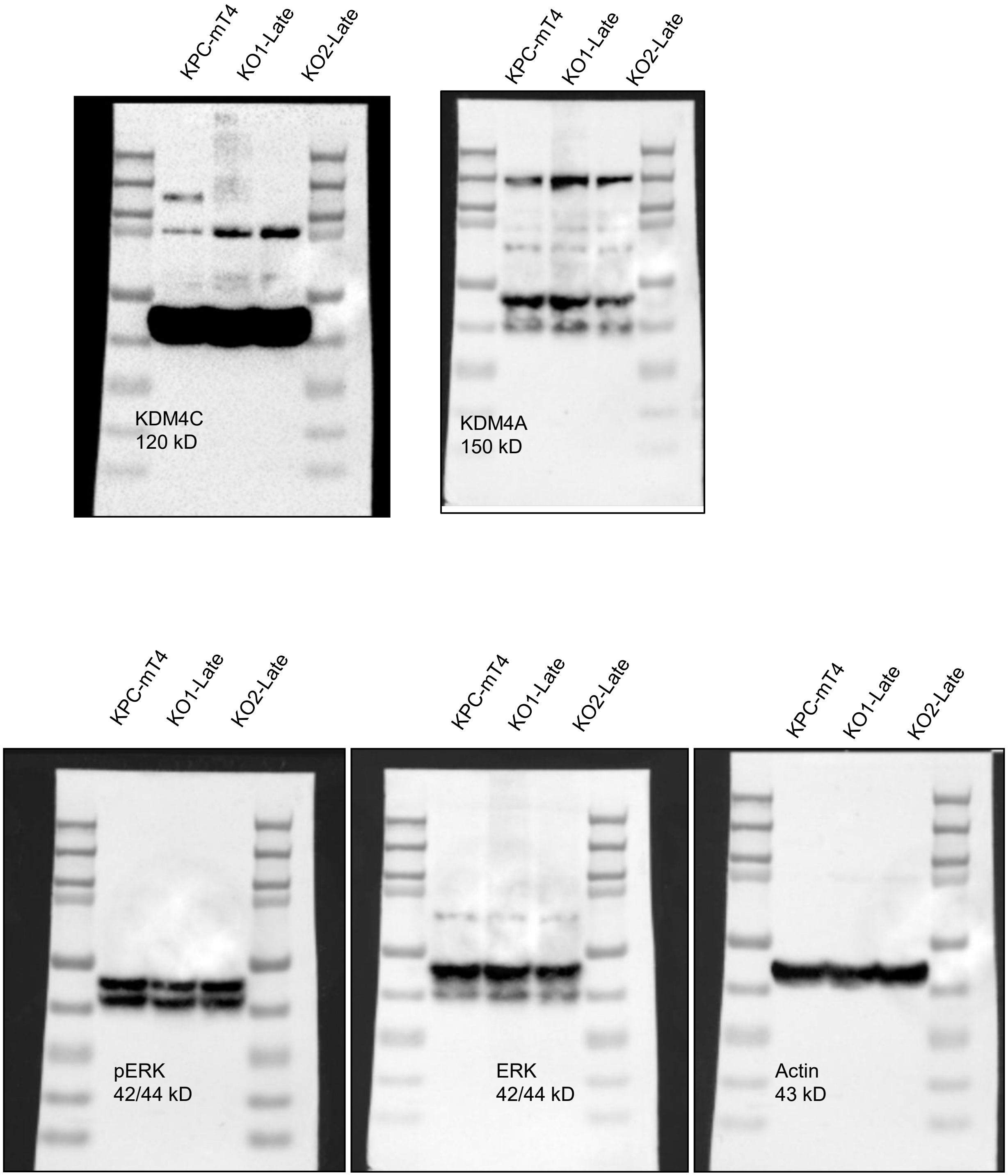
KPC-mT4
KO1-Late
KO2-Late
KDM4C
120 kD
KPC-mT4
KO1-Late
KO2-Late
KDM4A
150 kD
KPC-mT4
KO1-Late
KO2-Late
pERK
42/44 kD
KPC-mT4
KO1-Late
KO2-Late
ERK
42/44 kD
KPC-mT4
KO1-Late
KO2-Late
Actin
43 kD

Figure 5E

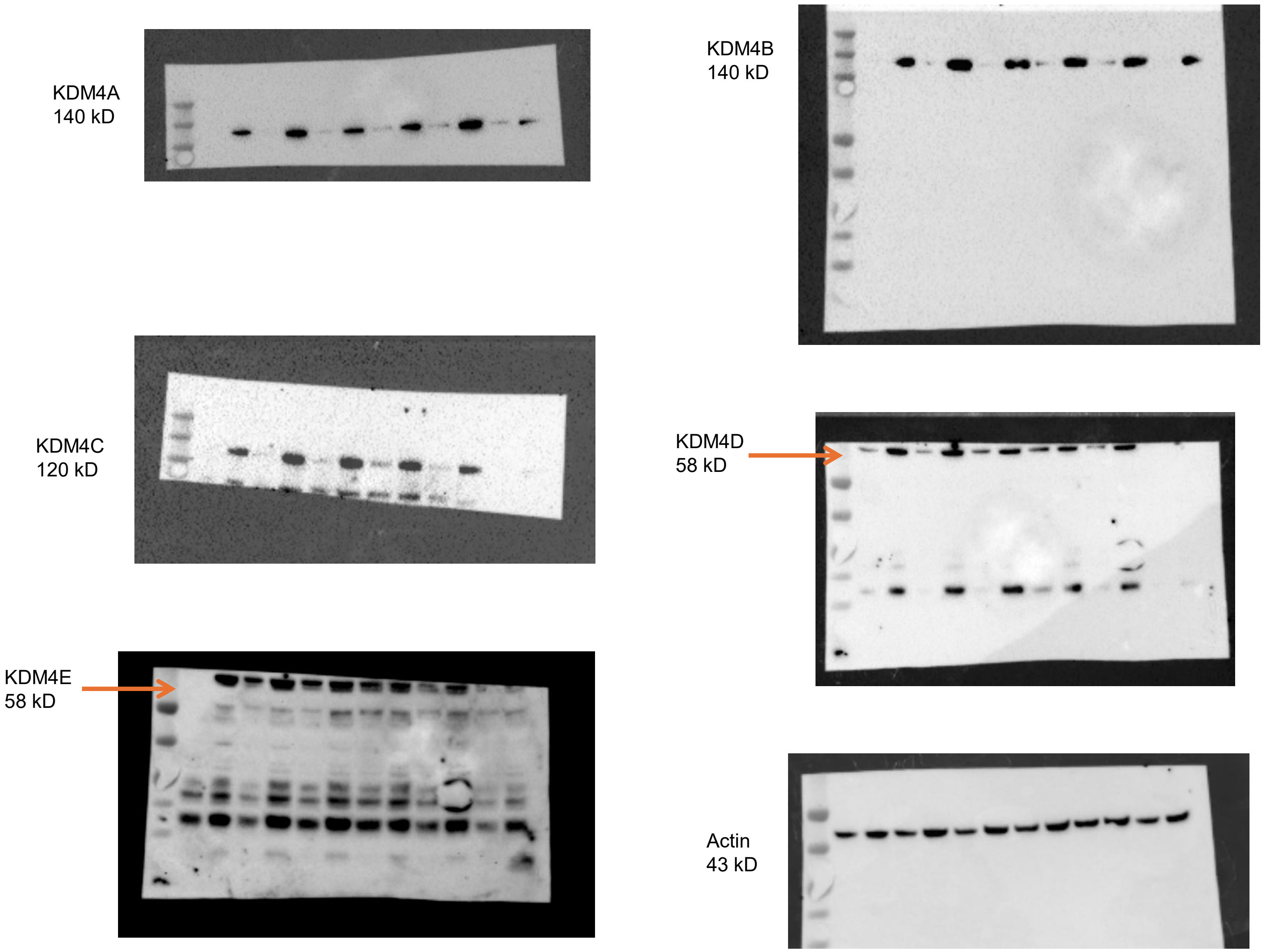

KDM4A
140 kD
KDM4B
140 kD
KDM4C
120 kD
KDM4D
58 kD
KDM4E
58 kD
Actin
43 kD

Figure 6A

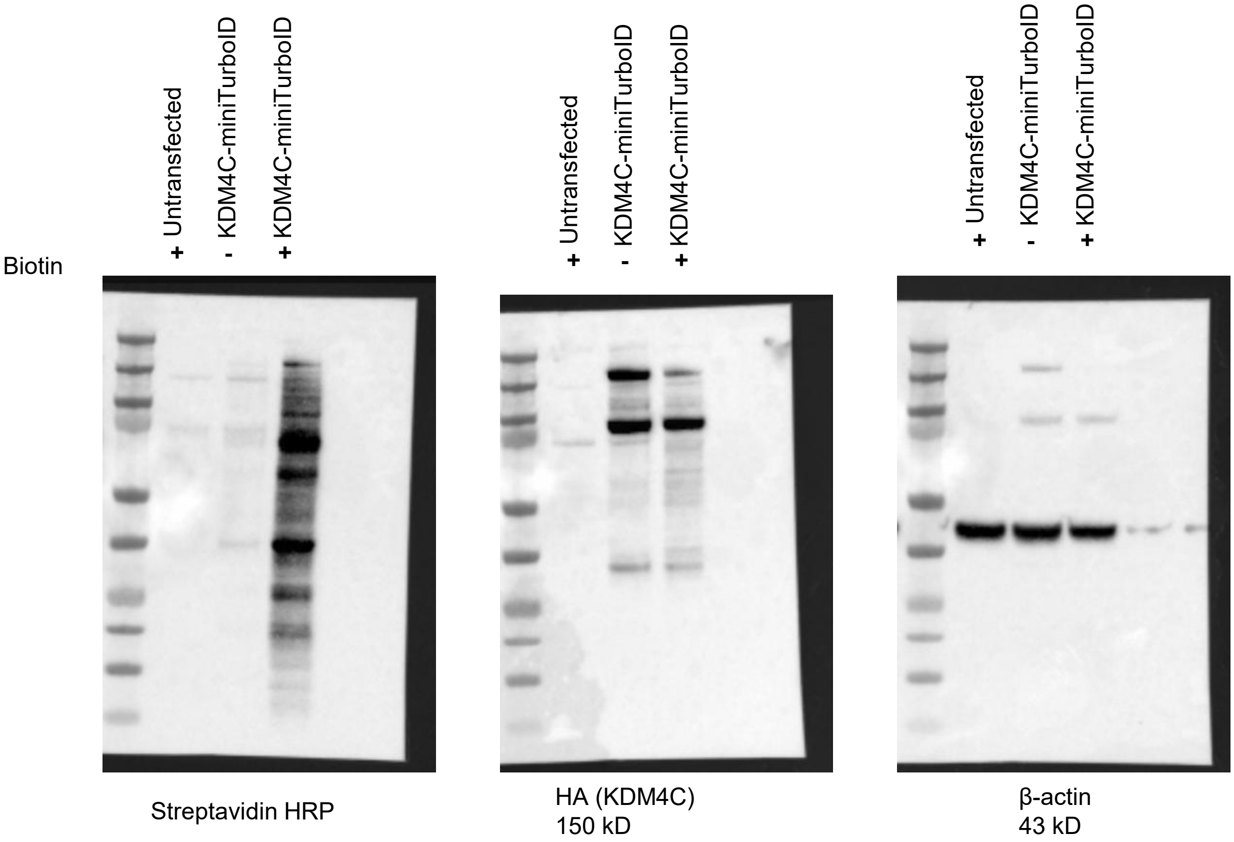

+ Untransfected
- KDM4C-miniTurboID
+ KDM4C-miniTurboID
Biotin
Streptavidin HRP
HA (KDM4C)
150 kD
β-actin
43 kD

Figure 6D

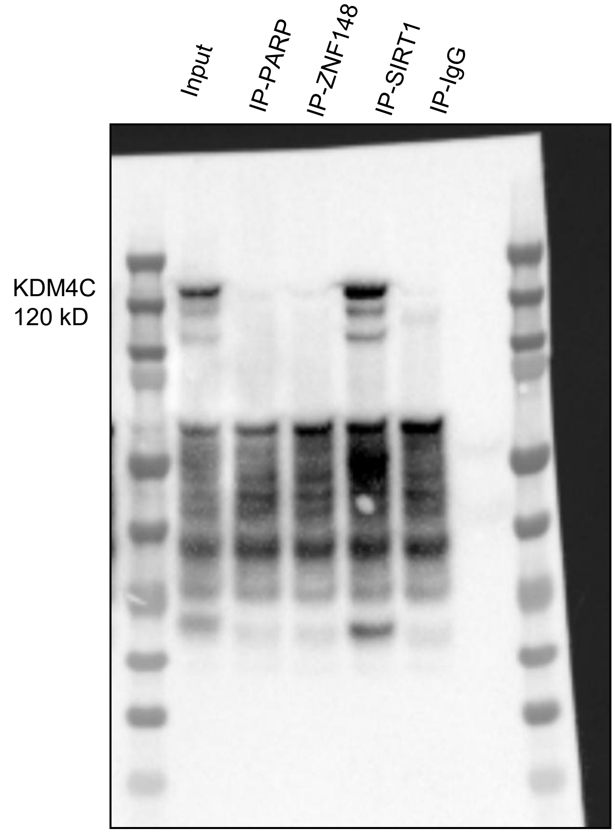


Figure 6F

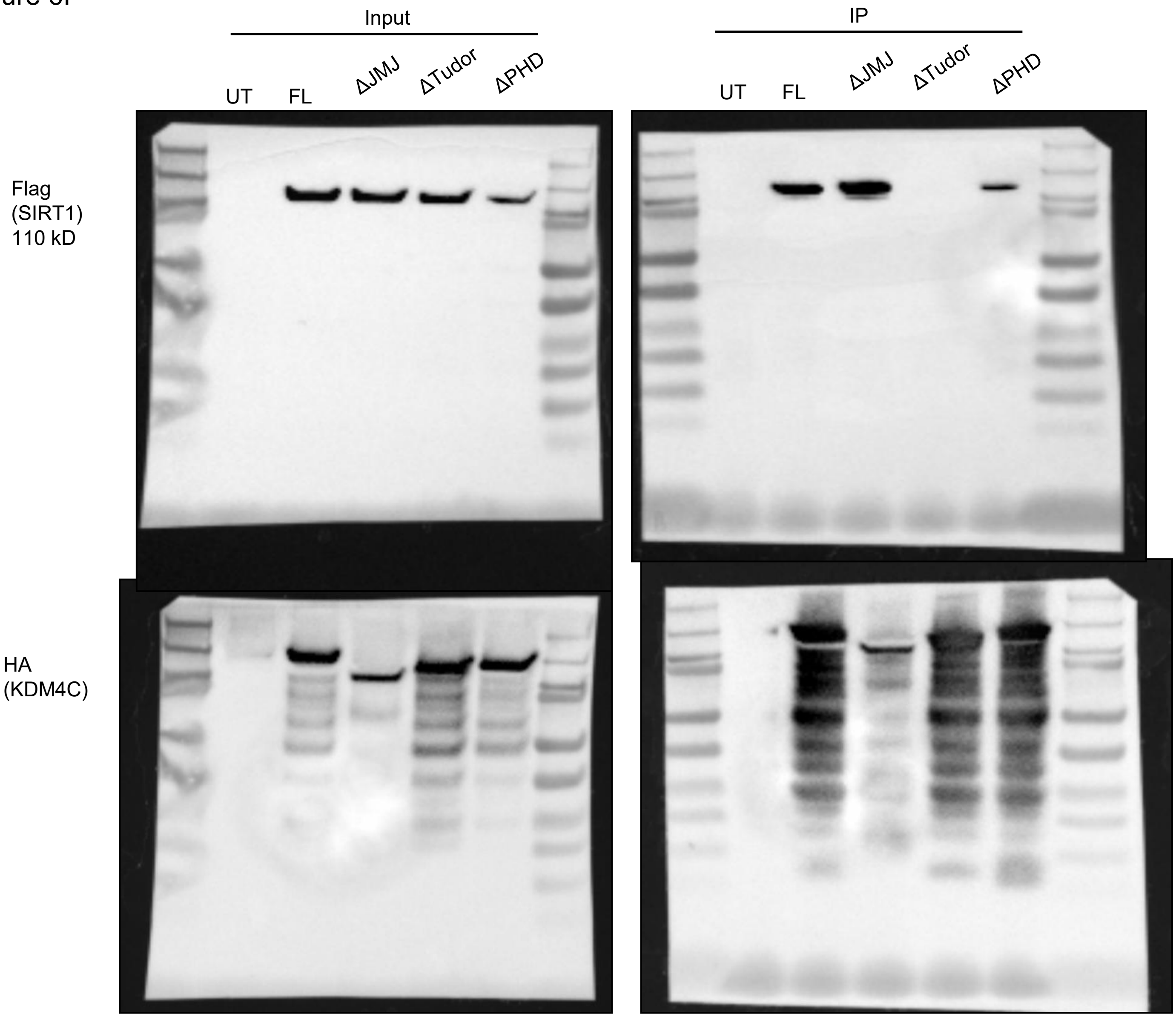

Figure 6I

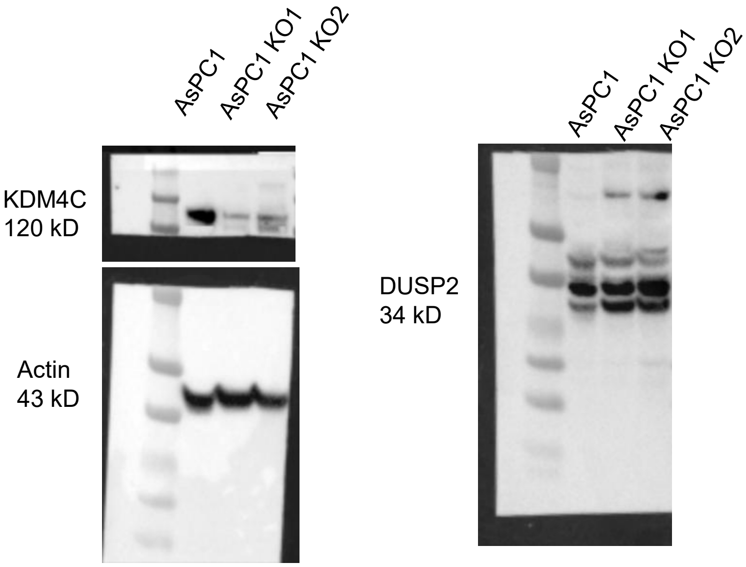
AsPC1
AsPC1 KO1
AsPC1 KO2
KDM4C
120 kD
Actin
43 kD
AsPC1
AsPC1 KO1
AsPC1 KO2
DUSP2
34 kD

Supplementary Figure 1A

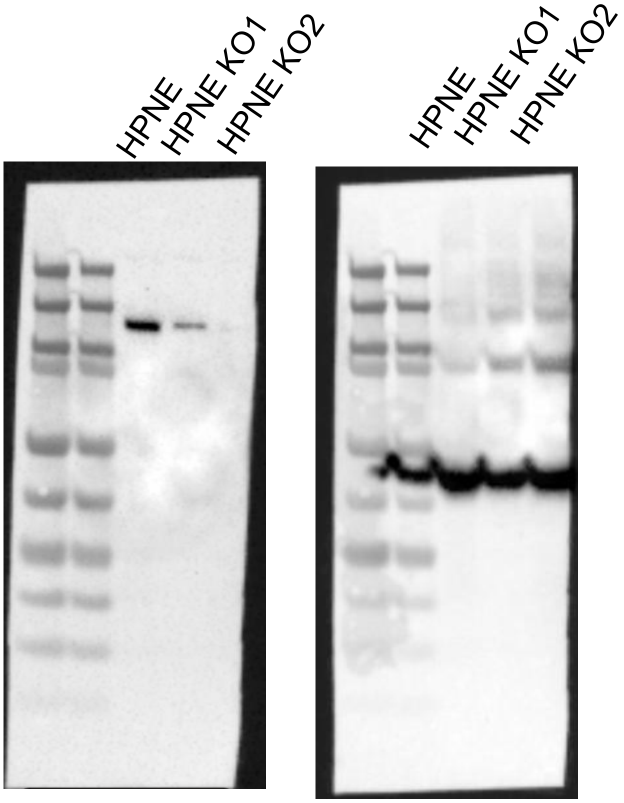


KDM4C
120 kD

β-actin
43 kD

Supplementary Figure 5A

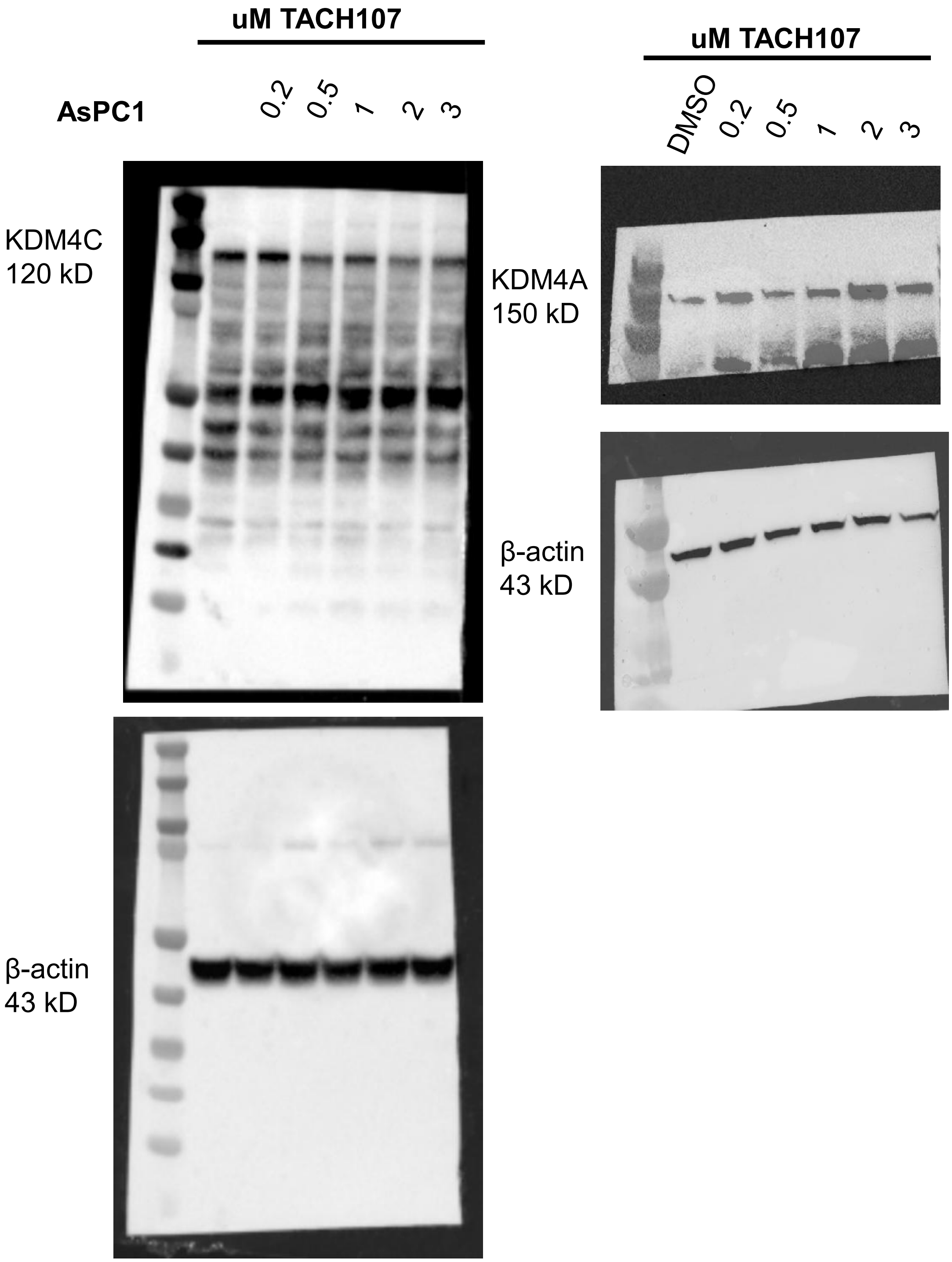

Supplementary Figure 6B

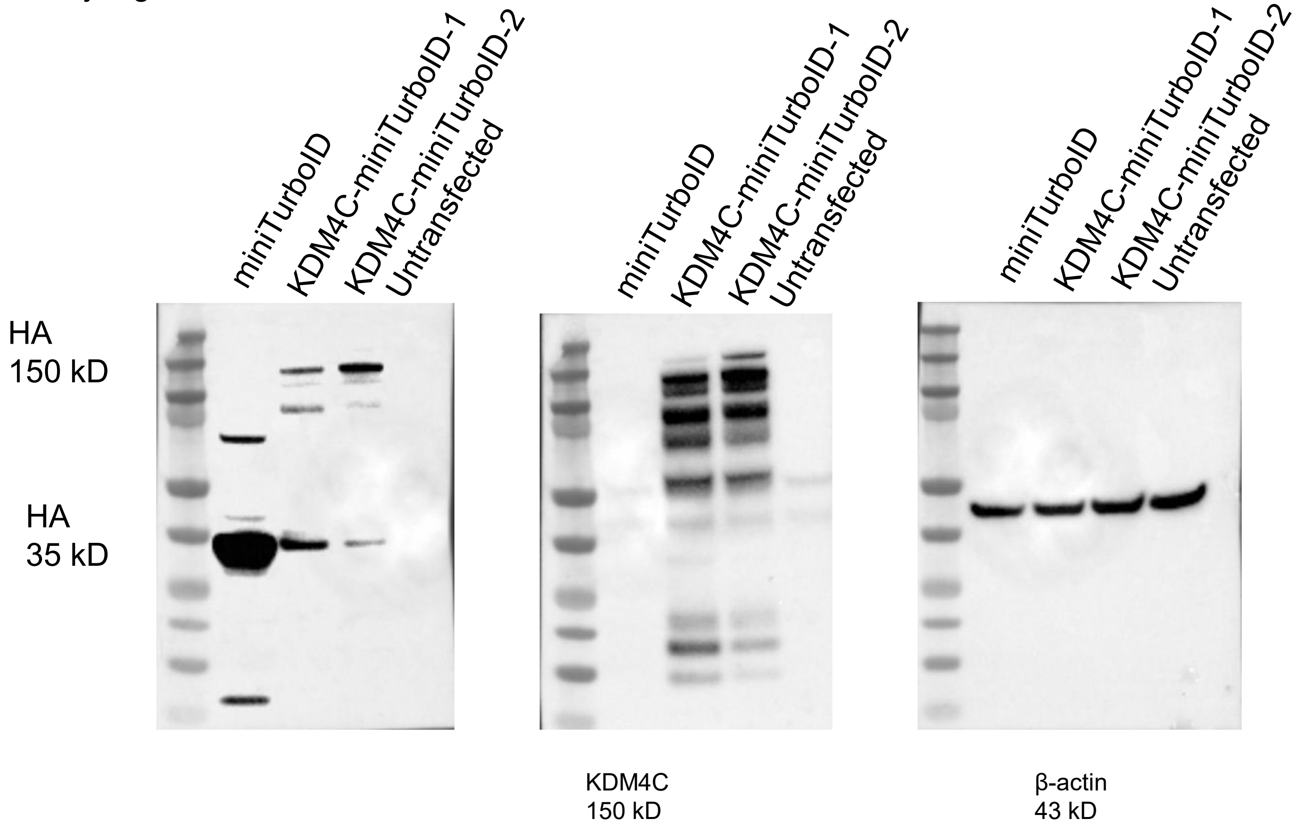

Supplementary Figure 6E

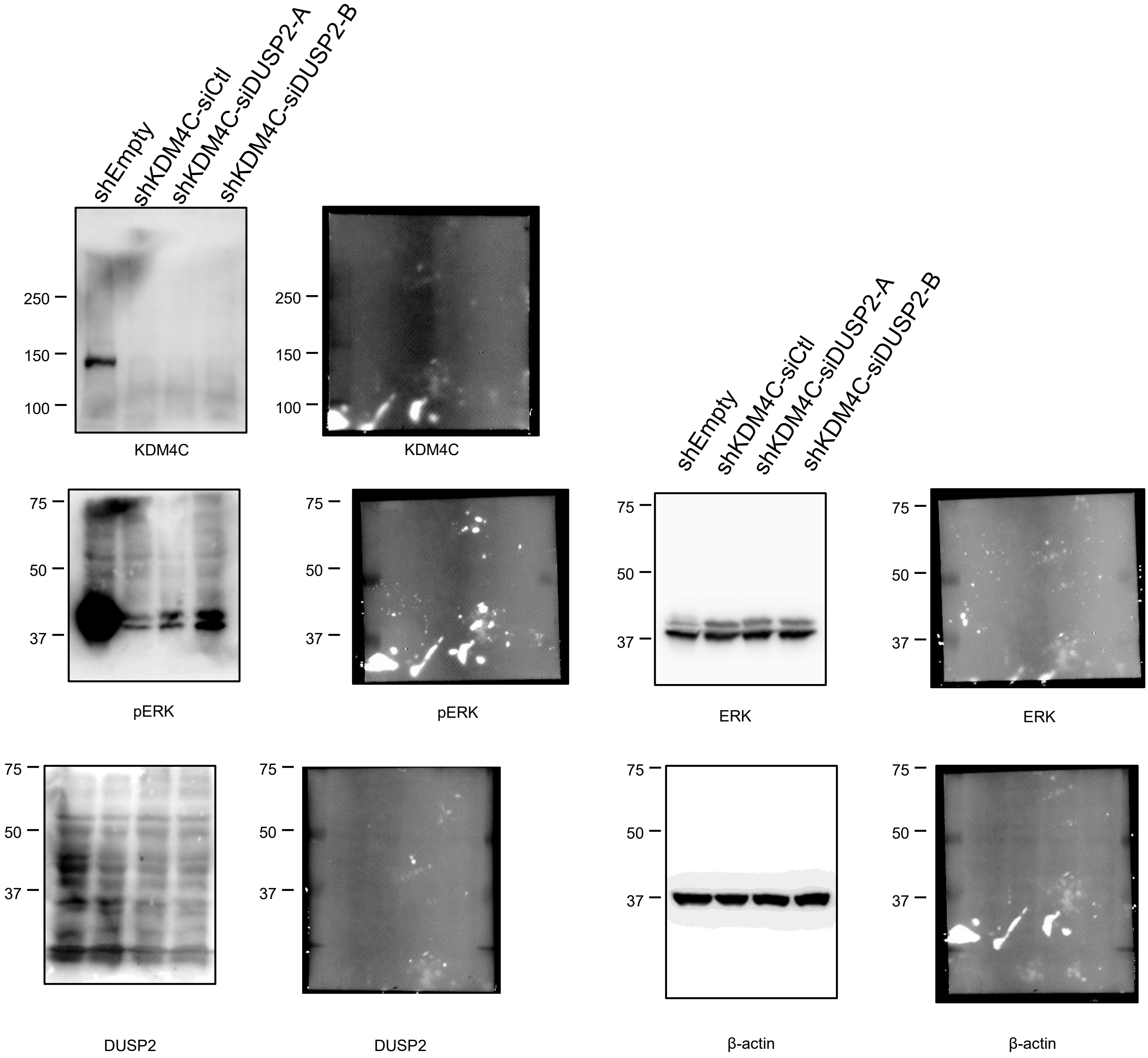

# Image Integrity Report

## Scan Information

Scan Date: 2025-12-26 09:34 UTC

Scanned file: KDM4C Manuscript- Supplemental Figures-revised 11.17.2025.pdf

## Assessment

**High Risk**

Imagetwin detected potential integrity issues with high confidence.

## Detection Summary

With the selected minimum confidence level of **33%**, the following potential integrity issues were detected:

| Integrity Issue | Count |
|---|---|
| AI image | 0 |
| Duplicates across publications | 0 |
| Duplicates in the scanned file | 11 |
| Splices | 2 |
| **Total** | **13** |

Comments from the authors are added to each detected issue below.

# Scanned File Preview

Below is an overview of the scanned file:

### KDM4C Manuscript- Supplemental Figures-revised 11.17.2025.pdf

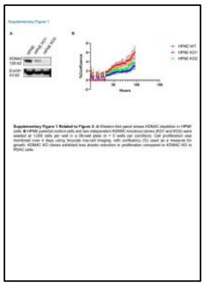

Page 1

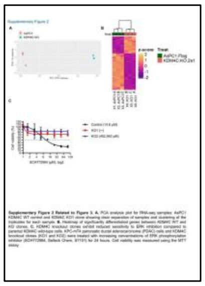

Page 2

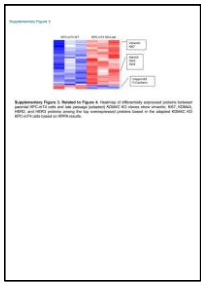

Page 3

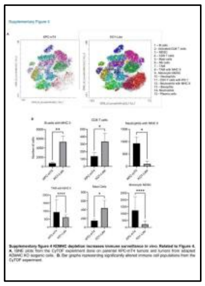

Page 4

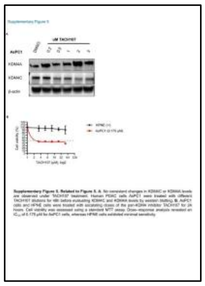

Page 5

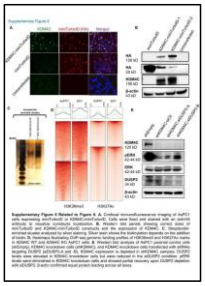

Page 6

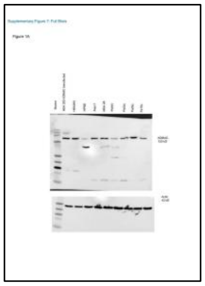

Page 7

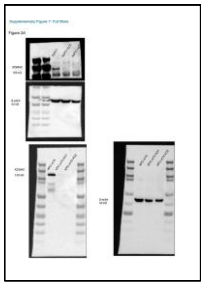

Page 8

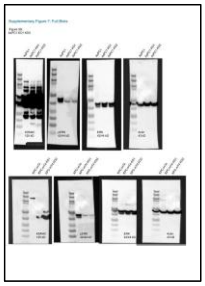

Page 9

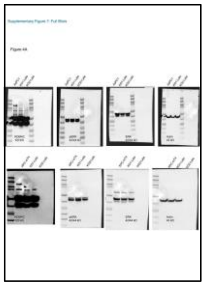

Page 10

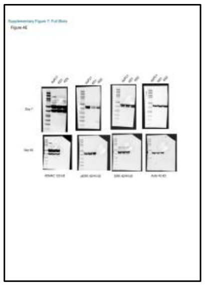

Page 11

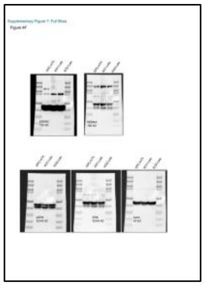

Page 12

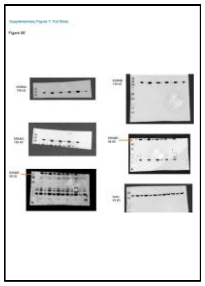

Page 13

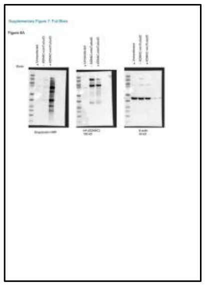

Page 14

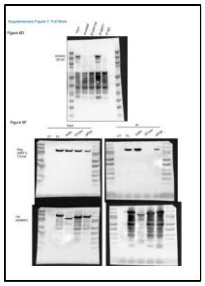

Page 15

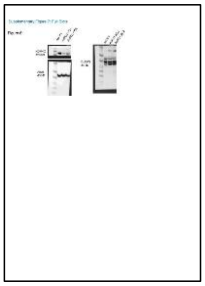

Page 16

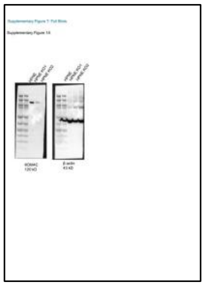

Page 17

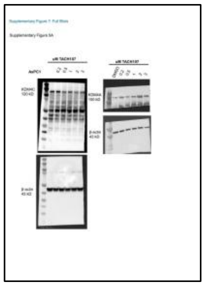

Page 18

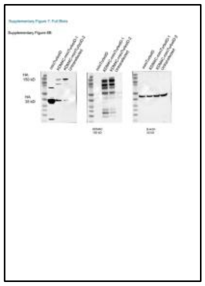

Page 19

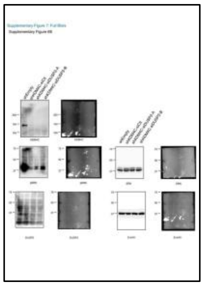

Page 20

# Overview of issue #1

Page 11 of KDM4C Manuscript- Supplemental Figures-revised 11.17.2025.pdf

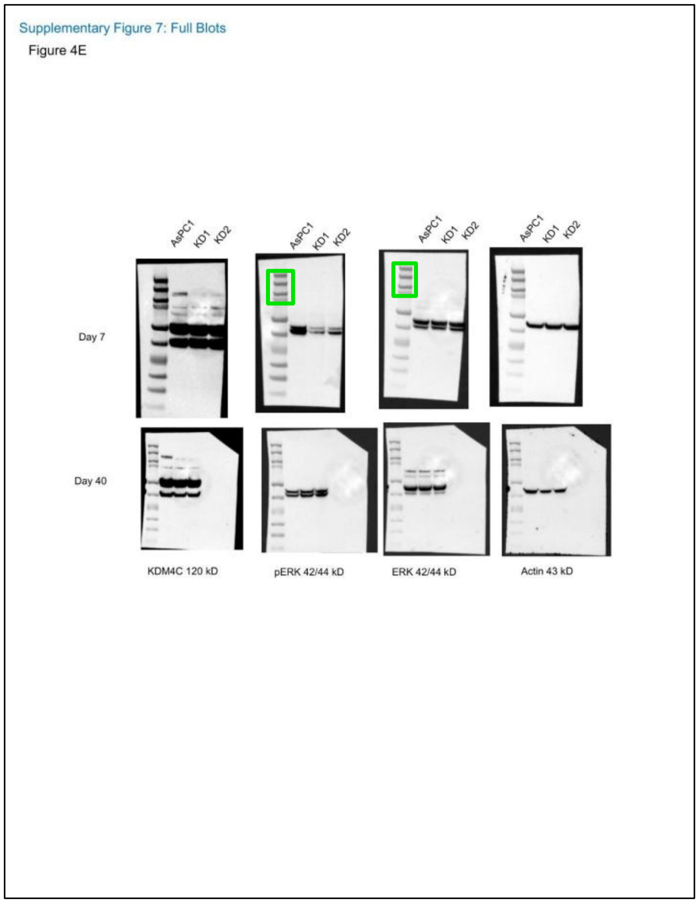


We detected 1 duplicate:

---

**Issue #1** | Duplicate | 52% confidence

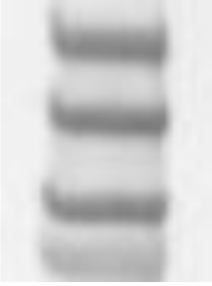

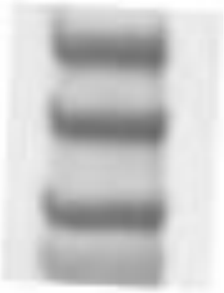

Same membrane, not a duplicate.

# Overview of issues #2-6

Page 12 of KDM4C Manuscript- Supplemental Figures-revised 11.17.2025.pdf

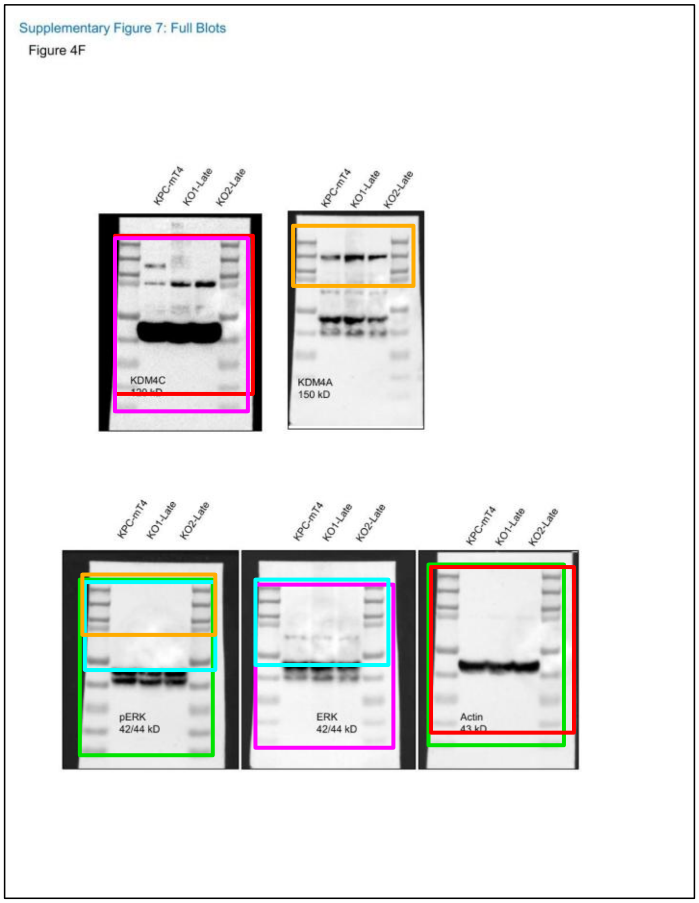


We detected 5 duplicates:

---

**Issue #2** | Duplicate | 87% confidence

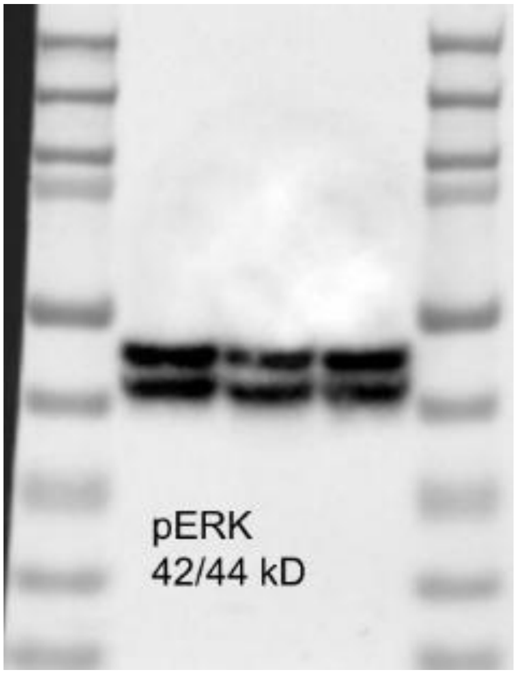


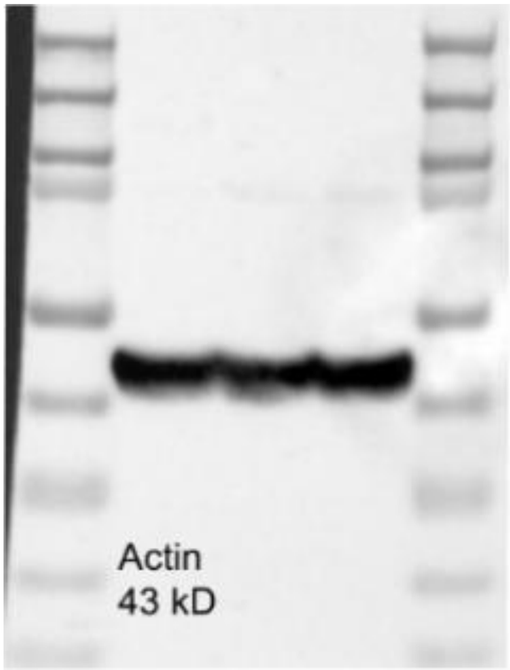


Same membrane, not a duplicate.

**Issue #3** | Duplicate | 79% confidence

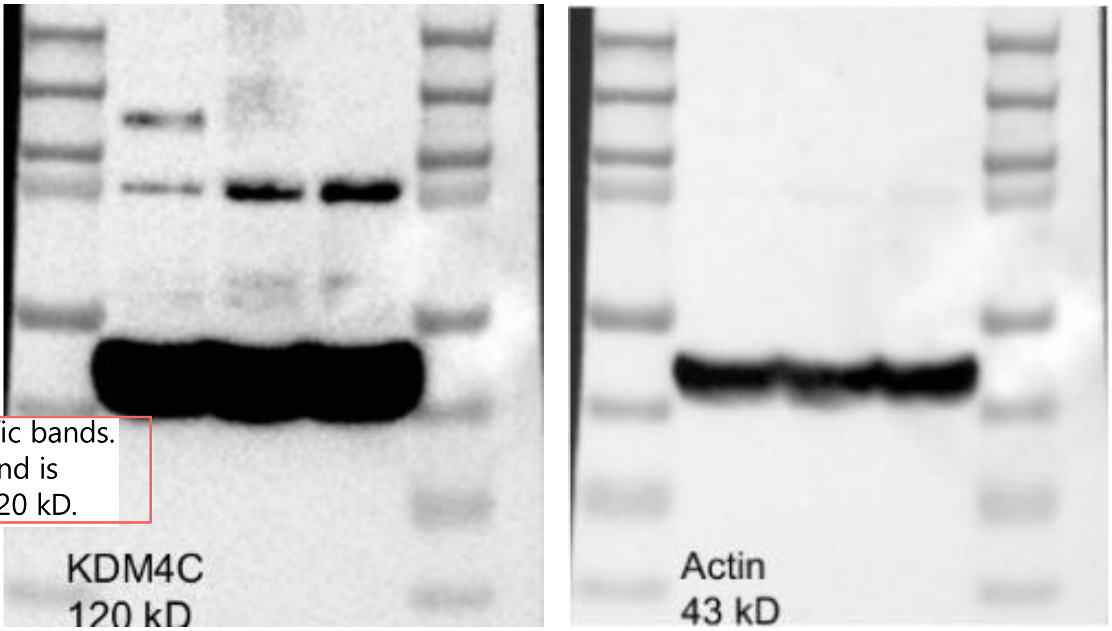


Non-specific bands. KDM4C band is visible at 120 kD.

---

**Issue #4** | Duplicate | 64% confidence

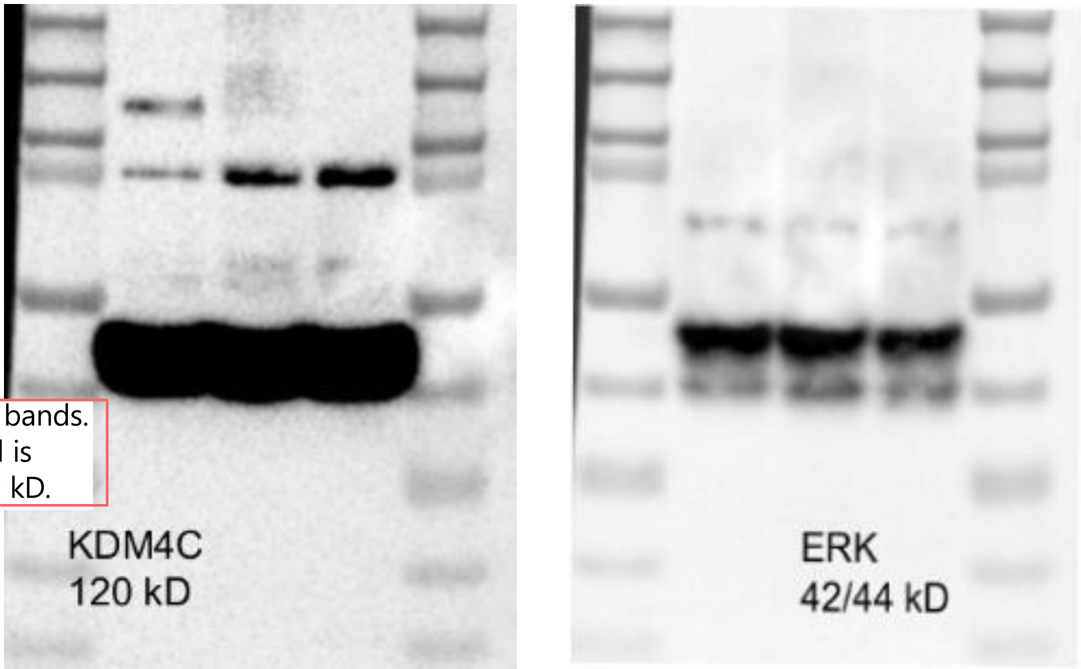


Non-specific bands. KDM4C band is visible at 120 kD.

**Issue #5** | Duplicate | 48% confidence

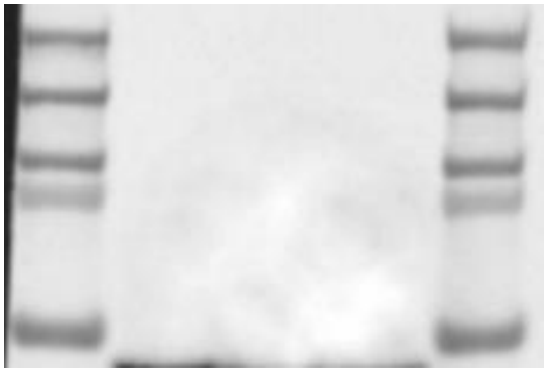

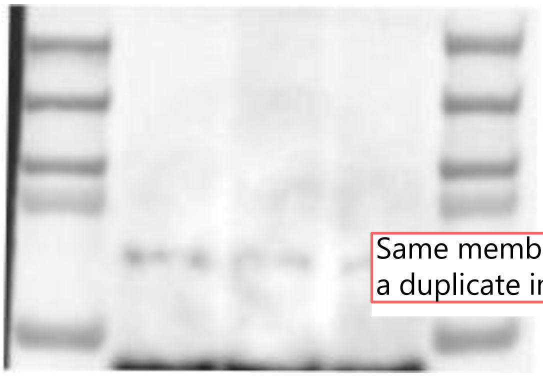

Same membrane. Not a duplicate image.

**Issue #6** | Duplicate | 44% confidence

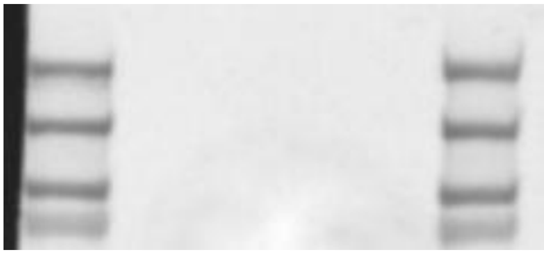

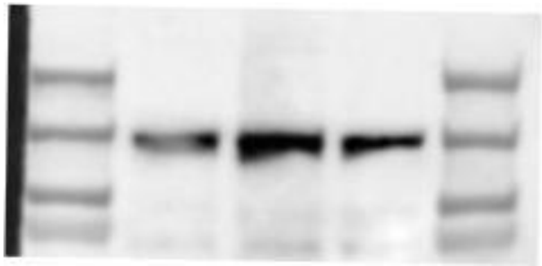

Same membrane. Not a duplicate image.

# Overview of issue #7

Page 5 of KDM4C Manuscript- Supplemental Figures-revised 11.17.2025.pdf

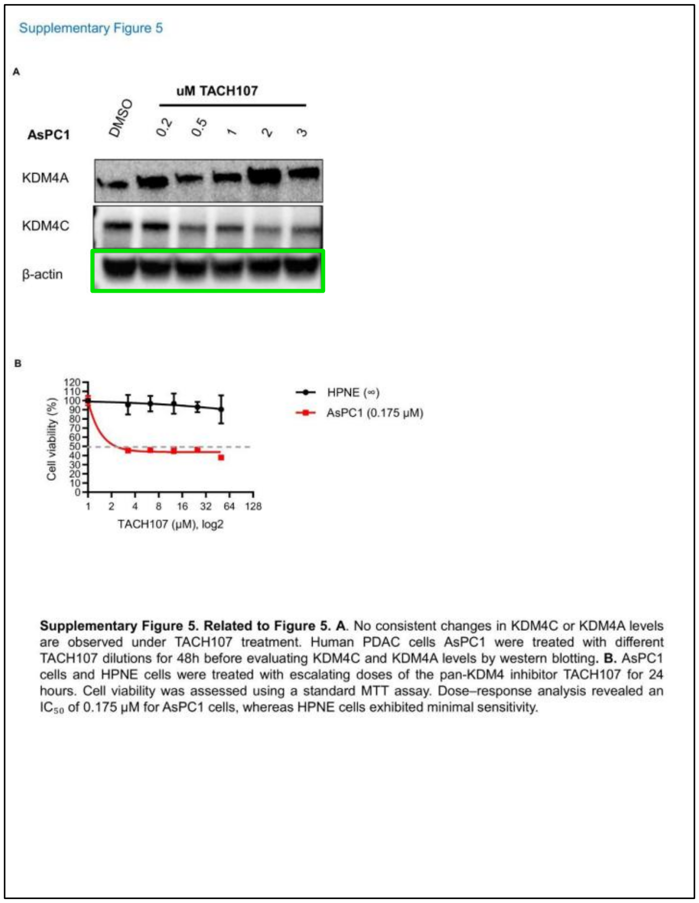


**Supplementary Figure 5. Related to Figure 5. A.** No consistent changes in KDM4C or KDM4A levels are observed under TACH107 treatment. Human PDAC cells AsPC1 were treated with different TACH107 dilutions for 48h before evaluating KDM4C and KDM4A levels by western blotting. **B.** AsPC1 cells and HPNE cells were treated with escalating doses of the pan-KDM4 inhibitor TACH107 for 24 hours. Cell viability was assessed using a standard MTT assay. Dose–response analysis revealed an $IC_{50}$ of 0.175 μM for AsPC1 cells, whereas HPNE cells exhibited minimal sensitivity.

We detected 2 splices:

---

**Issue #7-8** | Splices

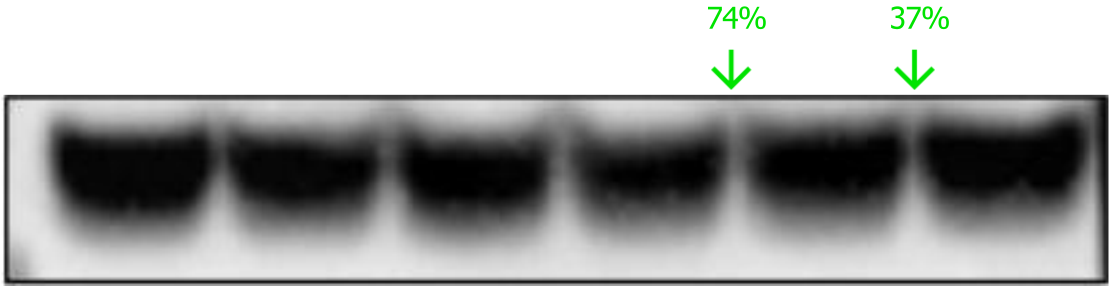


No splicing. Full blot image is included in Supplementary Figure 7.

# Overview of issue #9

Page 6 of KDM4C Manuscript- Supplemental Figures-revised 11.17.2025.pdf

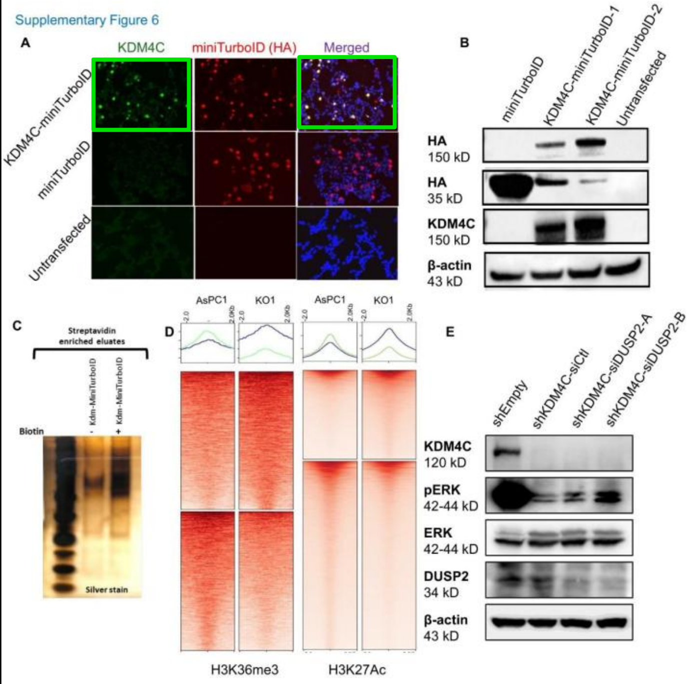


**Supplementary Figure 6 Related to Figure 6. A.** Confocal immunofluorescence imaging of AsPC1 cells expressing miniTurboID or KDM4C-miniTurboID. Cells were fixed and stained with an anti-HA antibody to visualize constructs localization. **B.** Western blot panels showing correct sizes of miniTurboID and KDM4C-miniTurboID constructs and the expression of KDM4C. **C.** Streptavidin-enriched eluates analyzed by silver staining. Silver stain shows the biotinylation depends on the addition of biotin. **D.** Heatmaps illustrating ChIP-seq genomic binding profiles of H3K36me3 and H3K27Ac marks in *KDM4C* WT and *KDM4C* KO AsPC1 cells. **E.** Western blot analysis of AsPC1 parental control cells (shEmpty), KDM4C knockdown cells (shKDM4C), and KDM4C knockdown cells transfected with siRNAs targeting DUSP2 (siDUSP2-A and -B). KDM4C expression is depleted in shKDM4C samples. DUSP2 levels were elevated in KDM4C knockdown cells but were reduced in the siDUSP2 condition. pERK levels were diminished in KDM4C knockdown cells and showed partial recovery upon DUSP2 depletion with siDUSP2. β-actin confirmed equal protein loading across all lanes.

We detected 1 duplicate:

---

**Issue #9** | Duplicate | 54% confidence

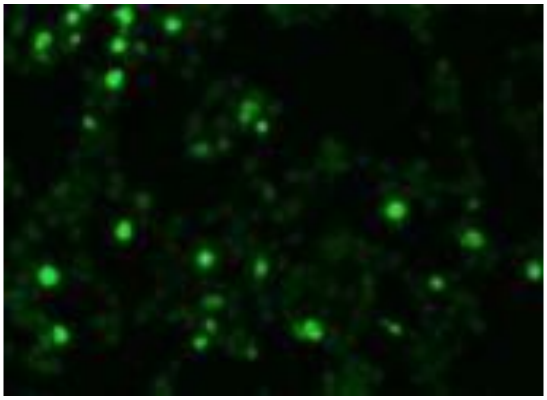

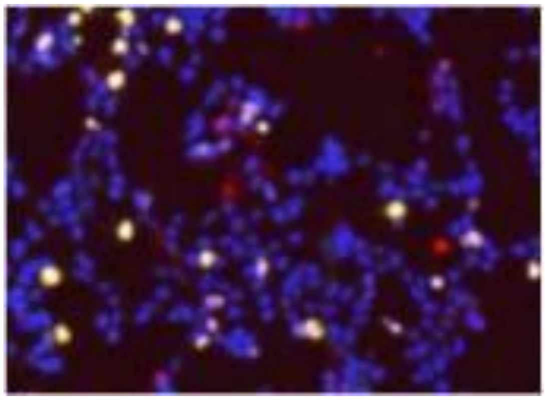

Merged image.

# Overview of issues #10-13

Page 6 of KDM4C Manuscript-Supplemental Figures-revised...

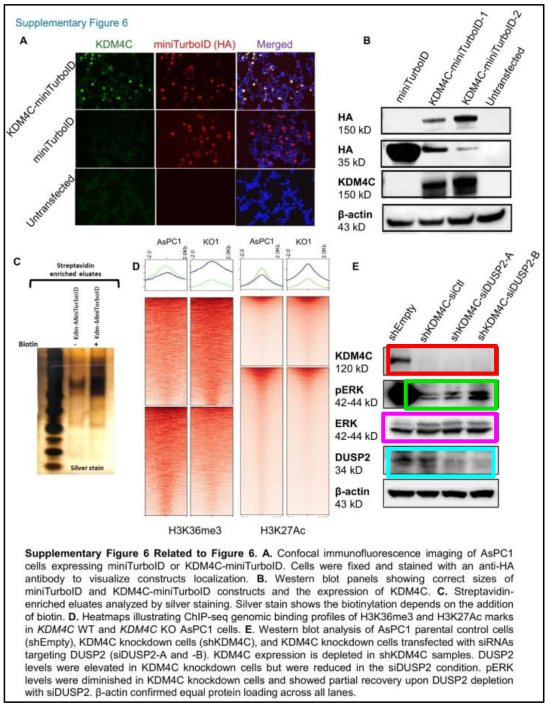


Page 20 of KDM4C Manuscript-Supplemental Figures-revised...

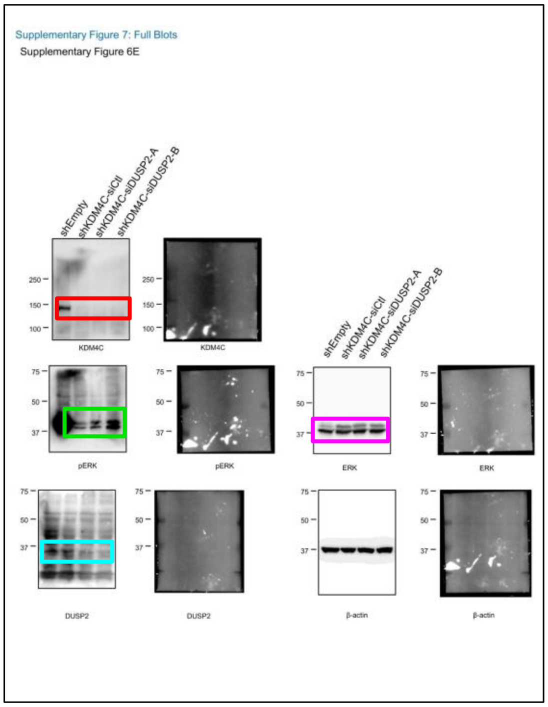


We detected 4 duplicates:

---

**Issue #10** | Duplicate | 99% confidence

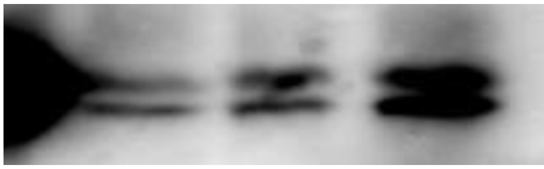

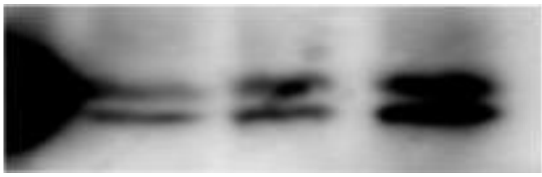

---

**Issue #11** | Duplicate | 99% confidence

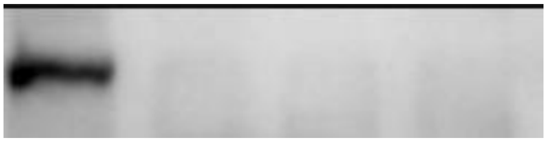

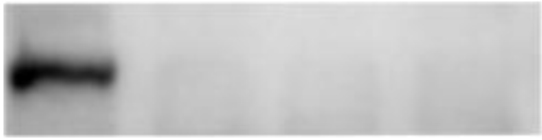

---

**Issue #12** | Duplicate | 99% confidence

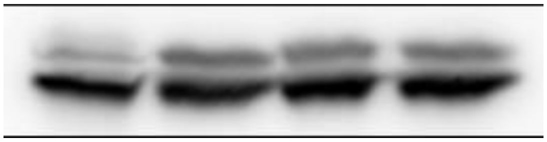

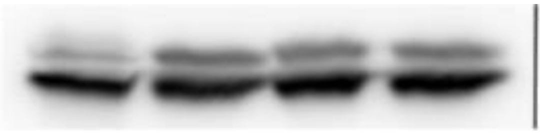

---

**Issue #13** | Duplicate | 99% confidence

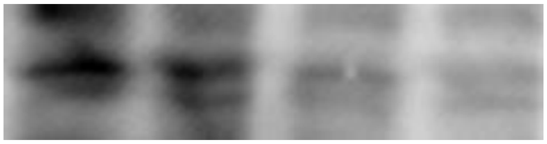

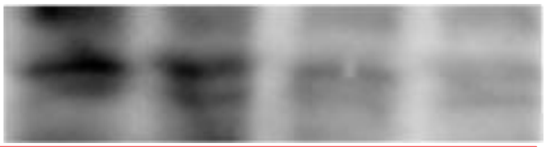

Same images shown cropped in the panel in Supplementary Figure 6E versus the full blot image in Supplementary Figure 7.

imagetwin

# Image Integrity Report

## Scan Information

Scan Date: 2025-11-16 18:53 UTC

Scanned file: KDM4C Manuscript- Main figures-revised.pdf

## Assessment

**High Risk**

Imagetwin detected potential integrity issues with high confidence.

## Detection Summary

With the selected minimum confidence level of **33%**, the following potential integrity issues were detected:

| Integrity Issue | Count |
|---|---|
| AI image | 0 |
| Duplicates across publications | 0 |
| Duplicates in the scanned file | 1 |
| Splices | 3 |
| **Total** | **4** |

Authors' comments are provided below each detected issue.

# Scanned File Preview

Below is an overview of the scanned file:

**KDM4C Manuscript- Main figures-revised.pdf**

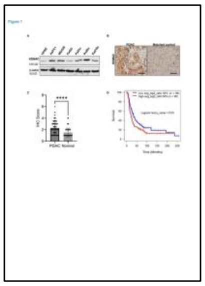
Page 1

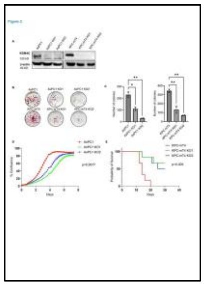
Page 2

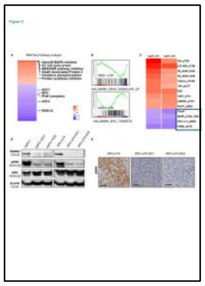
Page 3

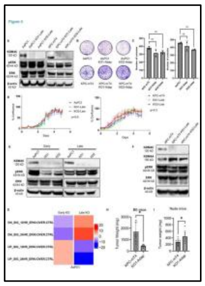
Page 4

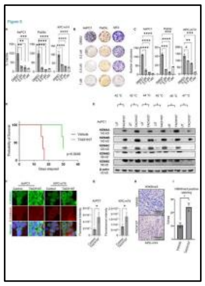
Page 5

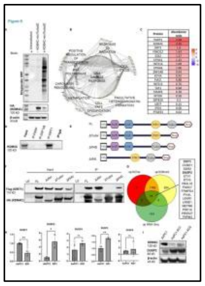
Page 6

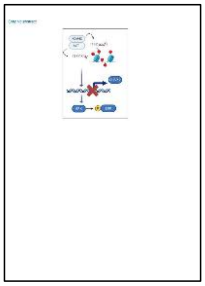
Page 7

# Overview of issue #1

Page 3 of KDM4C Manuscript- Main figures-revised.pdf

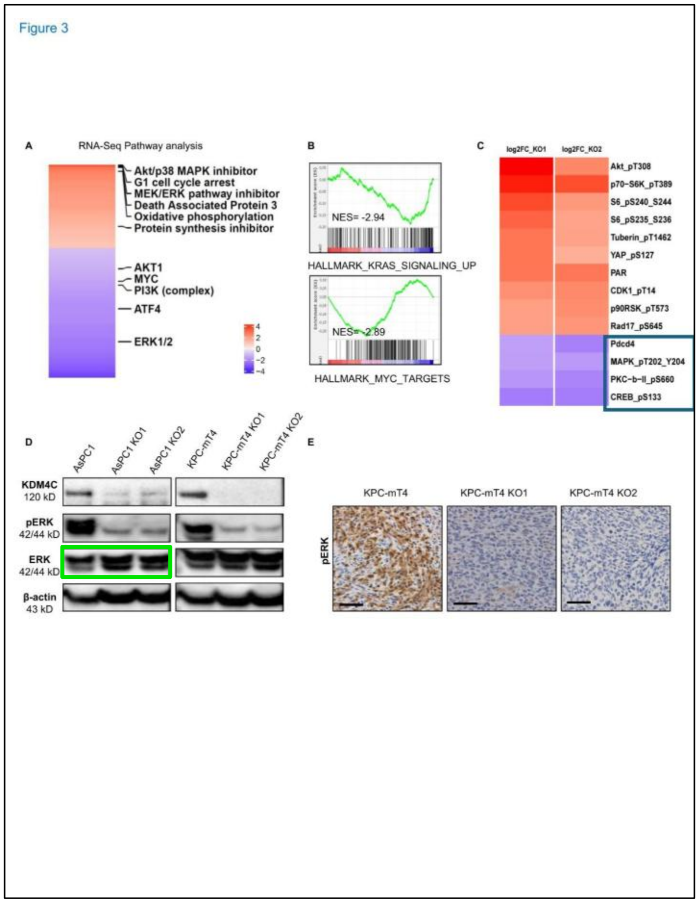


We detected 1 splice:

---

**Issue #1** | Splice

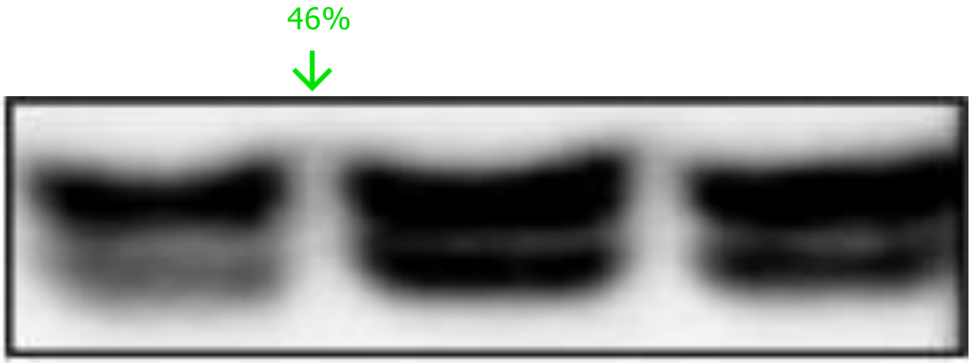


No splicing. Full blot image is provided in Supplementary Figure 7.

# Overview of issue #2

Page 4 of KDM4C Manuscript- Main figures-revised.pdf

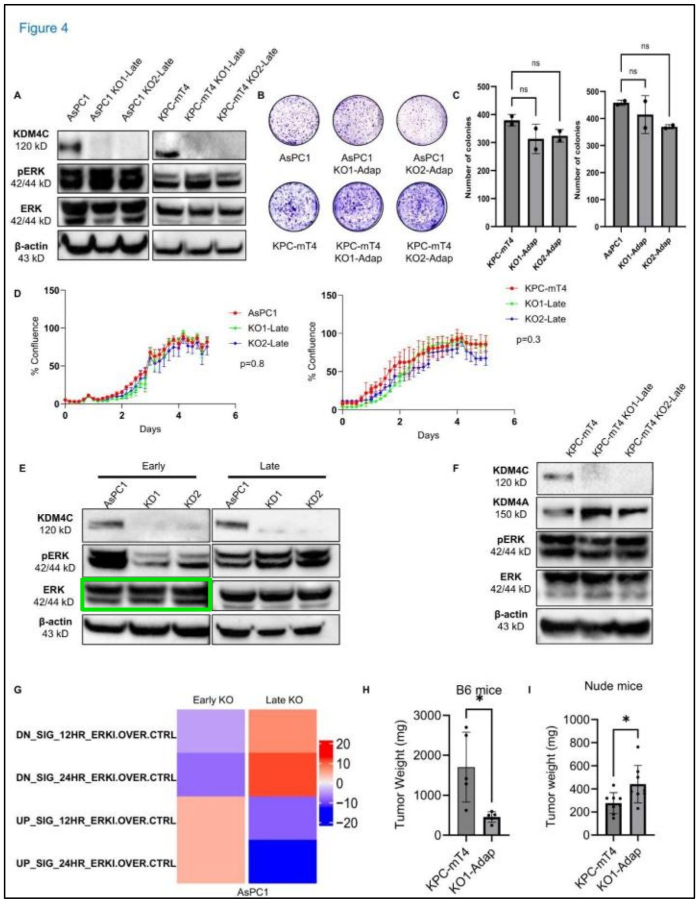


We detected 1 splice:

---

**Issue #2** | Splice

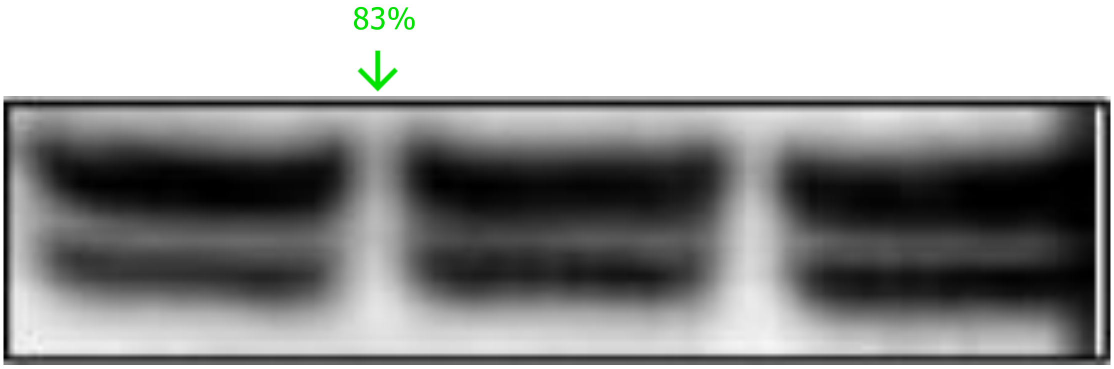


No splicing. Full blot image is provided in Supplementary Figure 7.

# Overview of issue #3

Page 5 of KDM4C Manuscript- Main figures-revised.pdf

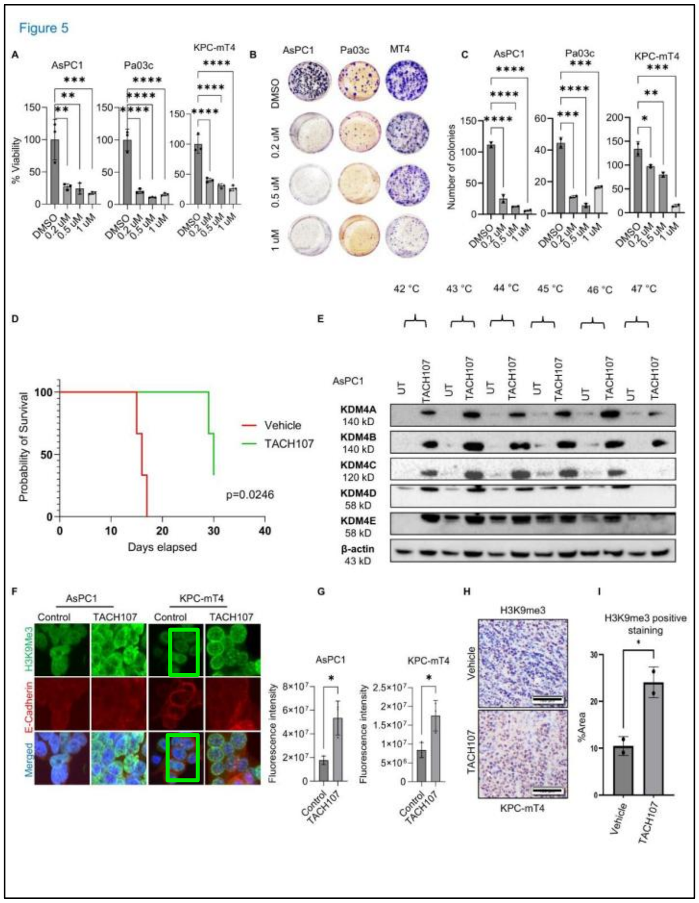


We detected 1 duplicate:

---

**Issue #3** | Duplicate | 36% confidence

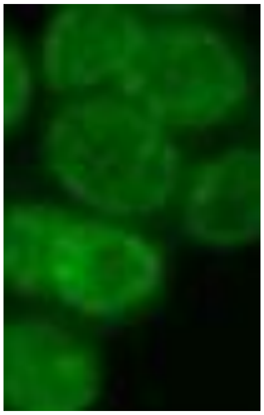

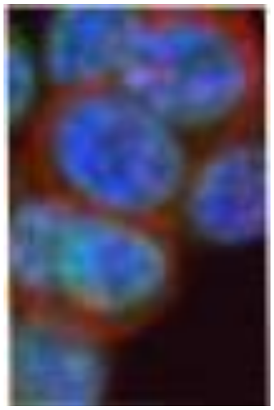

Merged image.

# Overview of issue #4

Page 6 of KDM4C Manuscript- Main figures-revised.pdf

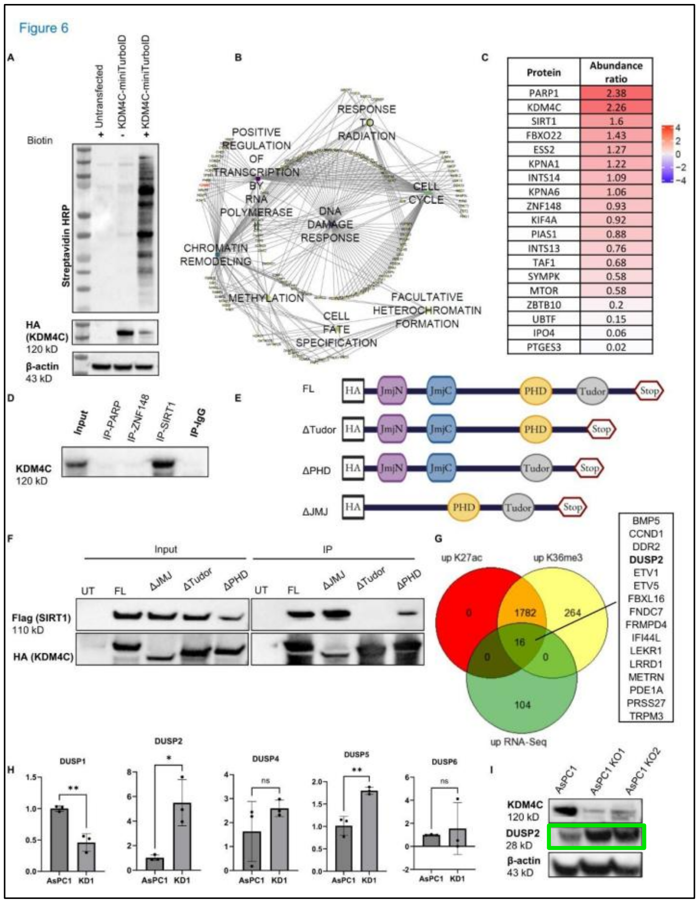


We detected 1 splice:

---

**Issue #4** | Splice

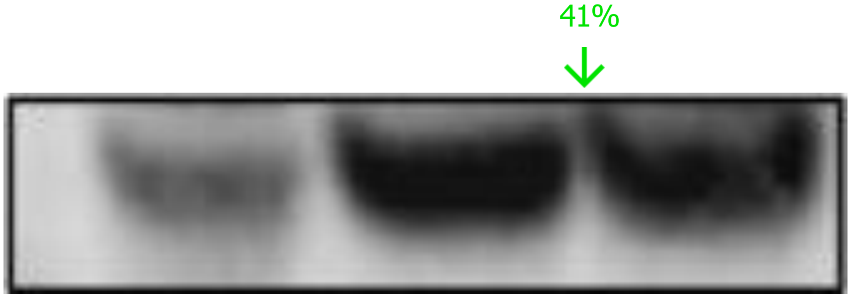


No splicing. Full blot image is provided in Supplementary Figure 7.

**Supplementary Figure 7** Uncropped western blot images for all western blot panels in the main and supplementary figures and the Imagetwin report with comments on detected issues.
